# A reciprocal relationship between markers of genomic DNA damage and alpha-synuclein pathology in dementia with Lewy bodies

**DOI:** 10.1186/s13024-025-00813-4

**Published:** 2025-03-20

**Authors:** David J. Koss, Olivia Todd, Hariharan Menon, Zoe Anderson, Tamsin Yang, Lucas Findlay, Ben Graham, Pawel Palmowski, Andrew Porter, Nicola Morrice, Lauren Walker, Johannes Attems, Simona S. Ghanem, Omar El-Agnaf, Fiona EN. LeBeau, Daniel Erskine, Tiago F. Outeiro

**Affiliations:** 1 Division of Neuroscience, School of Medicine, University of Dundee, Dundee, UK; 2 Translational and Clinical Research Institute, Faculty of Medical Sciences, Newcastle University, Newcastle, UK; 3 Newcastle University Protein and Proteome Analysis Unit, Newcastle University, Newcastle Upon Tyne, UK; 4 Neurological Disorders Research Centre, Biomedical Research Institute (QBRI), Hamad Bin Khalifa University (HBKU), Qatar Foundation, Doha, Qatar; 5 Faculty of Medical Sciences, Biosciences Institute, Newcastle University, Newcastle, UK; 6 Department of Experimental Neurodegeneration, Center for Biostructural Imaging of Neurodegeneration, University Medical Center Göttingen, Göttingen, Germany; 7 Max Planck Institute for Multidisciplinary Sciences, Göttingen, Germany; 8 Scientific Employee With an Honorary Contract at Deutsches Zentrum Für Neurodegenerative Erkrankungen (DZNE), Göttingen, Germany

**Keywords:** Dementia with Lewy bodies, Alpha-synuclein; DNA damage; synucleinopathy; Parkinson’s disease

## Abstract

**Background:**

DNA damage and DNA damage repair (DDR) dysfunction are insults with broad implications for cellular physiology and have been implicated in various neurodegenerative diseases. Alpha-synuclein (aSyn), a pre-synaptic and nuclear protein associated with neurodegenerative disorders known as synucleinopathies, has been associated with DNA double strand break (DSB) repair. However, although nuclear aSyn pathology has been observed in cortical tissue of dementia with Lewy body (DLB) cases, whether such nuclear pathology coincides with the occurrence of DNA damage has not previously been investigated. Moreover, the specific types of DNA damage elevated in DLB cases and the contribution of DNA damage towards Lewy body (LB) formation is unknown.

**Methods:**

DNA damage and aSyn pathology were assessed in fixed lateral temporal cortex from clinically and neuropathologically confirmed DLB cases and controls, as well as in cortical tissue from young 3-month-old presymptomatic A30P-aSyn mice. Frozen lateral temporal cortex from DLB and control cases was subject to nuclear isolation, western blotting, aSyn seed amplification and proteomic characterisation via mass spectrometry.

**Results:**

We detected seed-competent nuclear aSyn, and elevated nuclear serine-129 phosphorylation in DLB temporal cortex, alongside the accumulation of DSBs in neuronal and non-neuronal cellular populations. DNA damage was also present in cortical tissue from presymptomatic A30P mice, demonstrating it is an early insult closely associated with pathogenic aSyn. Strikingly, in postmortem DLB tissue, markers of genomic DNA damage-derived cytoplasmic DNA (CytoDNA) were evident within the majority of LBs examined. The observed cellular pathology was consistent with nuclear upregulation of associated DDR proteins, particularly those involved in base excision repair and DSB repair pathways.

**Conclusions:**

Collectively our study demonstrates the accumulation of seed-competent pathological nuclear associated aSyn, alongside nuclear DNA damage and the potential involvement of DNA damage derived cytoDNA species in cytoplasmic aSyn pathology. Ultimately, our study supports the hypothesis of a reciprocal relationship between aSyn pathology and nuclear DNA damage and highlights a potential underlying role for DNA damage in pathological mechanisms relevant to DLB, as well as other synucleinopathies, opening novel possibilities for diagnosis and treatment.

**Supplementary Information:**

The online version contains supplementary material available at 10.1186/s13024-025-00813-4.

## Introduction

The maintenance of accurate genetic information for the process of transcription and protein translation is essential for cellular homeostasis. Nevertheless, numerous DNA-damaging insults occur frequently, leading to a myriad of disruptive genomic alterations, including base modifications, single strand (SSBs) and double strand breaks (DSBs) [1, 2]. Thus, maintaining genomic integrity requires rapid engagement of DNA damage repair (DDR) pathways such as base-excision repair (BER), and DSB repair, via non-homologous end-joining (NHEJ) and homologous recombination (HR) [3]. Owing to the restriction of high-fidelity HR repair to specific cell cycle stages, the capacity of post-mitotic neurons to maintain genomic integrity is limited to the more error-prone NHEJ for DSB repair [4]. Consequently, aging neurons exhibit an increased number of individual genomic errors, leading to the accumulation of somatic mutations within the brain, compromising optimal neuronal function and increasing the risk for disease [5, 6].

Unresolved DNA damage not only impacts genomic integrity but can also drive cell death via apoptosis and parthantos, a form of cell death that occurs due to overactivation of the DNA repair enzyme, poly (ADP) ribose polymerase 1 (PARP1) [7]. Equally, through genomic fragmentation, DNA damage can generate various cytoDNA species, which are known to engage with pro-inflammatory pathways [8], but which may also, through aberrant protein-chromatin interactions, facilitate hallmark cytoplasmic protein aggregation [9–11].

Given the known vulnerability of neurons to accumulate age-associated DNA damage [12], and the overlap with neurodegenerative outcomes, it is unsurprising that DNA damage and genomic instability are emerging as cellular stressors central to the etiopathology of many age-related neurodegenerative diseases, including Alzheimer’s disease (AD), amyotrophic lateral sclerosis (ALS), and Parkinson’s disease (PD) [13–15].

Notably, recent evidence has highlighted a modulatory role of alpha-synuclein (aSyn), the main protein component of Lewy pathology in PD and Dementia with Lewy bodies (DLB), in DSB repair [15, 16]. aSyn is subject to post-translational modifications, such as phosphorylation at Serine 129 (pS129), ubiquitination, or acetylation [17]. aSyn modification may not only modulate it’s physiological roles but also may a lead to a loss of function and/or a gain of toxicity [18, 19]. Moreover, such pathological modification can result in the generation of seed competent species, which template aSyn misfolding, thereby contributing to the spreading of pathology during disease progression [19]. Critically, both pathological modification of aSyn and *SNCA* gene knockout have been associated with a reduced capacity for DSB repair [15, 16]. Consequently, excessive DSB and impaired DDR have been reported in various aSyn pathology models [15, 20–23], as well as the elevation of several forms of DNA damage in PD brain tissue [15, 24–27]. Strikingly, we recently demonstrated the occurrence of nuclear aSyn in postmortem human tissue and the occurrence of disease-dependent modifications of nuclear aSyn in DLB cases [28]. Nevertheless, the consequences of nuclear aSyn pathology upon genomic stability, DNA damage and the ensuing changes in nuclear proteome have yet to be determined in the context of DLB.

Here, employing fixed and frozen post-mortem human tissue of clinical and neuropathologically diagnosed cases of DLB, and an animal model of synucleinopathy, we investigated multiple aspects of DNA damage and nuclear pathology in relation to this disease.

## Materials and methods

### Human post-mortem brain tissue

Brain tissue samples from clinical and neuropathologically confirmed human cases of DLB and non-neurodegenerative disease controls (Con) were obtained from the Newcastle Brain tissue resource (NBTR). Slide mounted fixed paraffin embedded tissue sections from the middle and superior temporal gyrus (BA21/22) were used for immunohistochemistry (Con, n = 13 and DLB, *n* = 12). Frozen temporal cortex (BA21/22) from the contralateral hemisphere was used for subcellular fractionation and subsequent western blots (*n* = 14 for Con and *n* = 13 for DLB), seed amplification assay and ELISA (*n* = 4 for both) and for mass spectrometry (*n* = 9 for both).

Disease confirmation for each case was achieved by a review of clinical history upon death and detailed neuropathological assessment according to Braak Lewy body stages [29], the McKeith Criteria [30, 31] and the National Institute on Ageing -Alzheimer’s Association (NIA-AA) criteria [32] inclusive of Thal phases [33], Braak neurofibrillary tangle stages [34] and the Consortium to Establish a Registry for Alzheimer's Disease (CERAD) scoring for neuritic plaques [35]. Collectively there were no significant differences in either age, or post-mortem delay, between Con and DLB cases (*p* > 0.05). See Table 1 and Supplementary Table 1 for full details.

**Table 1:** Human tissue cohort

Disease	n	Sex (% male)	Age (years)	PMI (hrs)	NFT Braak stage	Thal Phase	CERAD	NIA-AA	LB Braak stage	McKeith Criteria
Histology a (b)										
Con	13 (12)	53.8.7% (58.3%)	60–99 82.38 ± 3.3 (60–95) (80 ± 3.2)	5–66 39.1 ± 5.7 (15–66) (42 ± 5.4)	0-III (0-III) 23.1 (25) %−0 53.8% (50) %-II 23.1 (25) %-III	0–5 (0–5) 46.1 (41.6) %−0 23.1 (25).%−1 7.7 (8.3) %−2 7.7 (8.3) %−3 7.7 (8.3) %−4 7.7 (8.3) %−5	neg-A (neg-A) 92.3 (91.7)%-neg 7.7 (8.3) %-A	no-inter 46.1 (41.6) %-no 46.1 (41.6) %-low 7.7 (8.3) %-inter	0 100%−0	No LB 100%-No LB
DLB	12	91.7%	71–91 78.5 ± 1.7	8–96 38.83 ± 8.8	II-III 41.7%-II 58.3% -III	0–5* 30%−0 10%−1 10%−2 20%−3 10%−4 20%−5	neg-B* 60%-neg 10%-A 30%-B	no-inter 20%-no 30% -low 50%- inter	4–6* 12.5% −4 87.5%−6	Limbic-Neo 12.5%-Limbic 87.5%-Neo
Western blot										
Con	14	58.3%	60–99 81.8 ± 3.1	5–74 41.7 ± 5.9	0-III 21.4%−0 50%-II 28.6%-III	0–5 50%−0 21.4%−1 7.1%−2 7.1%−3 7.1%−4 7.1%−5	neg-A 92.9%-neg 7.1%-A	no-inter 50%-no 42.9%-low 7.1%-inter	0 100%−0	No LB 100%-No LB
DLB	13	87%	71–91 78 ± 1.5	8–89 31.7 ± 6.9	II-III 38.57%-II 61.5%-III	0–5* 27.3%−0 9.1%−1 9.1%−2 18.1%−3 27.3–4 9.1%−5	neg-B 54.5%-neg 18.2%-A 27.3%-B	no-inter 18.2%-no 36.4%-low 45.4%-inter	6* 100%−6	Limbic-Neo 7.7%-Limbic 92.3%-Neo
SAA + ELISA										
Con	4	50%	73–95 82.3 ± 4.6	25–66 39.8 ± 9.7	0-III 25%−0 50%-II 25%-III	0–1 75%−0 25%−1	neg-A 75%-neg 25%-A	no-low 75%-no 25%-low	0 100%−0	No LB 100%-No LB
DLB	4	75%	72–91 79.3 ± 4.4	10–89 43 ± 17.1	III 100%-III	0–5 25%−0 25%−1 25%−3 25–5	neg-B 50%-neg 50%-B	no-nter 25%-not 25%-low 50%-inter	6 100%−6	Neo 100%-Neo
Mass spectrometry										
Con	9	66.7%	60–99 77.4 ± 4	5–74 41.67 ± 7.6	0-III 33.3%−0 44.4%-II 22.2%-III	0–5 33.3%−0 22.2%−1 11.1%−2 11.1%−3 11.1%−4 11.1%−5	neg 100%-neg	no-low 22.2%-not 77.7%-low	0 100%−0	No LB 100%-No LB
DLB	9	100%	71–82 77.4 ± 1.1	8–68 27.2 ± 6.6	II-III 55.6% -II 44.4%-III	0–4* 28.6%−0 14.3%−2 28.6%−3 28.6%−4	neg-B* 57.1%-neg 14.3%-A 28.6%-B	no-inter* 14.3%-no 42.9% -low 42.9%- inter	6* 100%−6	Limbic-Neo 11.1%-Limbic 88.9%-Neo

Human cases use for histology, western blot and mass spectrometry. Cases are separated by disease classification according to non-diseased controls (Con) and dementia with Lewy bodies (DLB) per methodological protocol (Histology, Western blot, ELISA + SAA and Mass spectrometry). Case numbers (n), sex, age, post-mortem interval (PMI), neurofibrillary tangle (NFT) Braak stage, Thal phase, Consortium to Establish a Registry for Alzheimer’s Disease (CERAD), the National Institute of Ageing – Alzheimer’s Association (NIA-AA) criteria, Lewy body (LB) Braak stage and McKeith criteria are provided. For age and PMI both range and mean ± SEM are provided. For numerical scores of pathology, range and percentage composition are given. For CERAD scores, negative (neg), A and B reported. For NIA-AA, not, low and intermediate (inter) risk for Alzheimer’s disease. For McKeith criteria, only percentage composition is given, where cases free of LBs (No LB), Limbic and neocortical (Neo) predominant are indicated. a = cohort for immunohistochemical assessment of γH2AX and XRCC1 and b = cohort for immunohistochemical assessment of γH2AX and nuclear pS129-aSyn. *=based on available data

### Animals

C57BL/6 wild type (WT) and A30P aSyn transgenic mice were used for immunohistochemical quantification of DNA damage. A30P mice express human mutant aSyn (A30P), under the control of the Thy-1 promotor [36], and were bred in-house from homozygous breeding pairs originally supplied by Dr. P. Kahle (University of Tubingen). Animals were rederived to a C57Bl/6 background via breeding with C57BL/6 mice (Charles River Laboratories, Tranent, UK) and maintained as previously described [37]. C57BL/6 WT control mice were used for comparison and purchased from Charles Rivers laboratories. In-house, as per the ARRIVE guidelines, all mice were group housed (3–4 mice per cage), on a 12 h light/dark cycle, lights on at 7:00 am, with food and water provided ad libitum.

Studies were conducted in tissue from 2.5–3 months of age (6–8 weeks of age), a time point in which A30P mice are pre-symptomatic and do not demonstrate robust PS129 aSyn pathology [38] but have been reported to show network excitability [37] and neuroinflammatory [39] changes in the hippocampus. All mice were anesthetised with isoflurane via inhalation, prior to intra-muscular injection of ketamine (> 100 mg/kg) and Xylazine (> 10 mg/kg) and euthanised by transcranial perfusion of 4% paraformaldehyde (PFA; Thermofisher). Fixed whole brains were removed, stored in PFA at 4^O^C overnight, before being transferred to cryoprotectant (30% glycerol and 30% ethylene glycol in 0.3M PBS in ddH_2_O) at 4^O^C overnight and finally stored at −20°C, prior to use. All experiments were performed in accordance with the UK animals (Scientific Procedures) Act 1986 and European Union directive 2010/63EU.

### Immunohistochemical staining of human post-mortem brain tissue

Paraffin embedded fixed slide mounted tissue Sects. (10 µm thick) were baked at 60°C for 30 min, dewaxed in Xylene (2 × 5 min) and rehydrated via submersion in descending concentrations of ethanol (99, 95 and 70%, for 5 min). Slides were washed in Tris-buffered Saline (TBS; 5 mM Tris, 145mM NaCl, pH 7.4) for 5 min, prior to antigen retrieval. Optimised nuclear staining as previously determined [28] was achieved via pressured heating in EDTA (1 mM, pH 8) for 2min, prior to submersion in formic acid (90%, 10 min) at RT. Slides were washed in TBS × 2 and in TBS with 0.1% Tween-20 (TBST) for 5 min, prior to being blocked in TBST containing 10% normal goat serum (NBS, Sigma) for 1 h at RT. Primary antibodies: mouse anti-γH2AX IgG1 (Clone JBW301, Cat# 05–636, Sigma, 1:500); rabbit anti-XRCC1 (Cat# HPA0061717, Sigma, 1:250) or rabbit anti-pS129-aSyn (EP1536Y, Cat# ab15253, abcam, 1:500) and mouse anti-NeuN IgG2b (Cat# ab104223, abcam, 1:250), were applied to slides in 10% NGS containing TBST overnight at 4^O^C. Following 3 × washes in TBST, slides were incubated in secondary antibodies; goat anti-mouse IgG1 Alexa 488 (1:500, Fisher Scientific), goat anti-mouse IgG2b Alexa 647 (1:500, Fisher Scientific) and goat anti-rabbit Alexa 594 (1:500, Fisher Scientific) in 10% NGS containing TBST for 1 h at RT. Following 3 × washes in TBST and 30s incubation in 70% ethanol, autofluorescence was quenched in 0.03% Sudan Black B in 70% ethanol for 5min. Stained slides were coverslipped with Prolong Diamond mountant with DAPI (Fisher Scientific) and stored in the dark at −20°C before being imaged.

Additional tissue punches as part of a tissue microarray (TMA) from the temporal lobe were stained for β-amyloid (Aβ) plaque, AT-8 phospho-tau and KM51 aSyn reactive Lewy neurite and LB pathology, as previously described [40]. Briefly, tissue Sects. (6 µm thick) were cut from paraffin tissue microarray blocks, containing cylindrical tissue cores taken from multiple brain regions. Sections were dewaxed and rehydrated in descending concentrations of ethanol (100, 95 and 70%, 5 min immersion). Following, antigen retrieval (for 4G8 amyloid plaques: submersion in 90% formic acid for 1h; for AT-8 phospho-tau: microwave-assisted antigen retrieval (800 W, 10min) in 10mM Citric acid, 0.05% Tween 20, pH6 and for KM51 Lewy pathology, pressure cooker heated for 2 min in 1mM EDTA, pH8), endogenous peroxidases were quenched (3% H_2_O_2_, 20 min submersion). Slides were incubated for 1h at RT in primary antibody staining ( 4G8, 1:16,000, Cat# 800,709, Biolengend, AT-8, 1:4000, Cat# AB_223647, Thermofisher, a-syn, 1:200 clone KM51, Cat# NCL-L-ASYN, Leica), visualised with MENAPATH HRP polymer detection kit (Menarini diagnostics, Wokingham, UK) and 3′3- diaminobenzidine DAB chromogen, prior to being co-stained with haematoxylin, prior to being dehydrated, cleared in xylene and mounted in dibutylphthalate (DPX).

Within the TMA slides, four tissue punches related to the lateral temporal cortex (BA21/22). Tissue punches were imaged at 100 X magnification (Nikon Eclipse 90i microscope, DsFi1 camera and NIS elements software V 3.0, Nikon), with multiple images taken to form a 3 × 3 grid of images, equating to 1.7mm^2^ (15% overlap between images). Following visual inspection, a region of interest (ROI) was established, excluding folds or tissue tears, from which consistent RGB thresholds were applied per stain across cases (4G8: R50–180, G20–168, B8–139, AT8: R25–170, G27–156, B11–126;, α-syn: R15–161, G7–139, B4–133) and area stained calculated from the resulting binary image. In addition to RGB thresholds, we set a restriction threshold for the assessment of 4G8 immunopositivity that excluded the measurement of immunopositive signals of a size below 100 μm2; this was necessary to ensure that physiological, cellular APP and or intracellular Aβ that is stained with 4G8 antibody was not included in the measurement For each case the mean of percentage area stained were used as a proxy of pathology burden.

### Immunohistochemical staining of mouse brain tissue

Fixed brains from WT (*n* = 5) and homozygous A30P (*n* = 5) brains from 2.5–3-month male old mice were removed from cryoprotectant solutions, hemisected and cut into 35 µm thick sections by means of a freezing microtome and stored in 1 M PBS at 4°C prior to staining.

Free-floating brain sections were washed three times in 1 M PBS and subsequently blocked for five hours submerged in 10% NGS containing 1 M PBS with 1% triton (PBS-T), on a vibrating (160 rpm) shaker (Grant – bio PMS- 1000i) at RT. Sections were then incubated in 10% NGS PBS-T with primary antibodies for XRCC-1, γH2AX and NeuN (as above) overnight at 4^O^C with continued shaking (160 rpm). Following 3 times washes in PBS at RT, sections were incubated with appropriate secondary antibodies (as detailed above) for 3 h (RT, 160 rpm). Autofluorescence was then quenched, incubating sections in 70% ethanol for 5 min, prior to submersion in 0.03% Sudan black B in 70% ethanol for 5 min and washed 3 times in PBS. The last PBS wash contained the nuclear acid Hoechst 33,342 dye (2 µg/ml). Sections were then transferred directly to superfrost plus slides and mounted in Fluoromout-G (Thermofisher) and allowed to dry overnight. Stained slide mounted sections were stored in the dark at −20^O^C, prior to being imaged.

### Nuclear immunofluorescence tissue staining

Immunofluorescence-stained post-mortem human and mouse brain tissue slides were imaged either on a widefield fluorescence (Nikon Eclispse 90i microscope, DsQ1Mc camera and NIS element software V 3.0, Nikon) or confocal (Leica SP 8, LAS Z software, Lecia -microsystems) microscope.

Quantifications of XRCC-1 and γH2AX in human and mouse tissues as well as γH2AX and pS129-aSyn in human sections were conducted via Leica SP 8 confocal microscope under 63 × oil immersion lens. In human slides, 5 fields of interest were selected at random from the lateral temporal grey matter (cortical layers V-VI) and 3 fields of interest were selected at random in mouse cortical sections (Somatosensory cortex). Per region, Z-stacks (21 images at 0.3 µm step thickness, totalling a depth of 6.3 µm) were captured for XRCC-1, γH2AX, NeuN immunoreactivity and DAPI or Hoechst fluorescence for each sample (For XRCC-1 and γH2AX, laser setting in human: 12.1%, 405 nm, 14.2% 488 nm, 1.4% 552 nm 3.2% 638 nm and in mouse: 8% 405 nm; 30.5% 488 nm; 25% 552 nm and 2.3% 638 nm and for γH2AX and pS129-aSyn in human: 7.6% 405 nm; 8% 488 nm; 8.6% 552 nm and 1.9% 638 nm).

For imaging, Z-stacks were summated using Z-project maximum intensity function of ImageJ (NIH), DAPI fluorescence was used to manually trace individual nuclei as regions of interest (ROIs), and corresponding intensity of NeuN staining within each ROI used to divide ROIs into NeuN positive (NeuN + , neuronal cells) and NeuN negative (NeuN-, non-neuronal cells). On occasion, some images contained overt staining of the blood vessels, these were readily identified with a distinct staining pattern were excluded from the analysis. Mean fluorescence intensity values for γH2AX, XRCC-1 and pS129 aSyn, without threshold application, were calculated per ROIs and pooled into either NeuN + and NeuN- generating a mean value per image per immunoreactive channel. Values per image were then further averaged to determine a mean value of fluorescence per channel for NeuN + and NeuN- nuclei per case. The resulting values were then pooled into disease conditions or genotype prior to comparative analysis. Disease or genotype comparisons (e.g. Con Cf. DLB and WT cf. A30P) of channel fluorescence as well as raw counts NeuN + and NeuN- nuclei number where performed via Mann–Whitney tests (GraphPad Prism, Ver 5). For mouse comparisons, owing to the existence of a clear outlier in the A30P group, a Grubbs outlier test was conducted to statistically determine deviation from the group mean, accordingly 1 A30P mouse was excluded from analysis (see Supplementary Fig. 4 for inclusive data). Correlative analysis was also performed between mean fluorescence for pS129 and γH2AX, and between yH2AX and TMA derived % area coverage of hallmark burdens as was as with neuropathological scores in human cases via Spearman’s correlation (Prism). Additionally, frequency plots of immunoreactivity per channel were calculated, based on all sampled ROIs per disease condition or genotype as per NeuN + and NeuN- nuclei (Prism). Such quantification was done to visualise alterations in nuclear immunoreactivity at a cellular population level per disease condition or genotype.

### Quantification of genomic DNA damage derived chromatin in Lewy bodies

Sections from DLB cases, either stained with γH2AX and pS129 aSyn or with 53BP1 (Rabbit anti-53BP1, 1:250, Cat# 4937S, Cell Signalling) and pS129 (staining protocol as outlined above) or pS129 aSyn and H3K27me3 (Mouse anti-histone H3 -tri methyl K27, 1:200, Cat# mabcam 6002, abcam) were imaged on a widefield microscope with a 20 × objective lens or confocal with × 63 objective lens. For the quantification of γH2AX positive LBs, one section (from block I as outlined previously [41], mounted on a 76 × 39 mm slide, including BA21, 22, 41 and 42) from 10 DLB cases and for H3K27me3 positive LBS, 8 DLB cases were visually inspected for all pS129 immunoreactive LB and co-localisation of γH2AX/H3K27me3 determined. For all cases, no less than 25 LBs per section were sampled. Percentage LBs γH2AX/H3K27me3 + ve and γH2AX/H3K27me3-ve were plotted as cumulative bar charts to establish a measure of the frequency of genomic DNA damage containing LBs within DLB cases. γH2AX/H3K27Me3 colocalization within LBs was further validated by confocal imaging at 63 × oil immersion objective lens and Z-stack imaging.

### Subcellular fractionation of frozen post-mortem human tissue

Nuclear isolation was performed as previously reported [28]. In brief, ~ 250 mg of frozen lateral temporal cortex from Con and DLB cases (*n* = 9) were homogenised 1:16 (W:V) in nuclear extraction buffer (0.32 mM sucrose, 5 mM CaCl_2_, 3 mM Mg(Ac)_2_, 10 mM Tris–HCl, 0.1% NP-40, pH 8) supplemented with cOmplete proteases inhibitor cocktail and phostop tablets (1 per 10 ml, Sigma) via a dounce homogeniser (50 manual strokes per sample). Nuclei were then isolated from the resulting crude whole tissue homogenate via sucrose gradient centrifugation (1.8 M Sucrose, 3 mM Mg(Ac)_2_, 10 mM Tris–HCl, pH 8, 107,000 rcf × 2.5 h, 4^O^C). Nuclear pellets were washed twice in 0.01 M PBS prior to being resuspended in 500 µl 0.01 M PBS, recovered and stored at −80°C prior to use.

### Seed amplification assay and ELISA

Nuclear fractions from Con (*n* = 4) and DLB (*n* = 4) cases were pretreated with DNAse (5 u/ml, 15 min RT, RQ1 DNAse, Promega) and adjusted to 0.1 mg/ml prior to use in an aSyn seed amplification assay (SAA). SAAs were performed as previously outlined for brain homogenate, with each case run in triplicate [42]. Briefly, wild-type monomeric aSyn was passed through a 100-kD molecular weight cut-off filter and added at a concentration of 0.1 mg/ml to reaction mixture (0.1 M piperazine-N, N9 bis (ethanesulfonic acid), 0.5M NaCl, 10 µM Thioflavin T (ThT), p.h 6.5). Immediately following aSyn addition, 160 µl of reaction mixture was added to wells of a 96-well clear bottomed black plate (Thermo fisher) and 40 µl of adjusted nuclear extracts added to test wells, prior to sealing the plate. Plates were subsequently incubated (37°C, 120 h) in an Omega FLUOstar plate reader, with intermittent shaking (1 min 500 rpm every 15 min). Over the course of the 120 h incubation period, ThT fluorescence measurements (450 nm excitation and 480 nm emission) were taken every 45 min and 5 µl samples of reaction products were taken at 20, 60 and 120 h.

Collected reaction product were analysed in triplicate using an in-house aSyn-O2 antibody based aSyn oligomer specific ELISA. The aSyn-O2 antibody is a conformation specific antibody with high affinity for aSyn aggregation and has previously been validated in ELISA measures of human brain tissue and in the SAA production of cytoplasmic brain fractionates [43, 44] (antibody available upon request to Prof. Omar El-Agnaf). Accordingly, 96 well plates were bound with capture antibody Syn-O2 (0.5 µg/ml, overnight, 4°C), blocked for 1 h and samples diluted in 50% RIPA buffer (1:1000) incubated for 1 h at 37°C. Bound oligomers were subsequently labelled with a biotinylated Syn-O2 detection antibody (0.5 µg/ml; 1h incubation at 37°C) prior to the addition of HRP-conjugated Streptavidin (1:5,000, 30 min, 37°C) and resulting chemiluminescence measured via a ParkinElmer Envision plate reader.

Quantification of SAA was determined via a 2-way- repeated measures ANOVA with time and disease as variables. Quantification of aSyn oligomeric ELISA with a 2-way ANOVA with time and disease as variables and a subsequent time point comparisons conducted via Bonferroni post-test. For all tests *p* < 0.05 was taken as significant.

### Western blot analysis

Nuclear fractions from Con (*n* = 14) and DLB (*n* = 13) were pretreated with DNAse (50 u/ml, 15 min RT, RQ1 DNAse, Promega), were adjusted to 80 ng/µl with LDS sample buffer (Fisher Scientific), reducing agent (Fisher Scientific) and dH_2_O as per BCA protein concentration assay (Fisher Scientific). Prepared samples were heated at 70°C for 10 min and loaded at 4 µg/lane and separated via SDS page (4–12% Bis–Tris gels, Fischer Scientific) with MES buffer (200 V, 35 min) and transferred to nitrocellulose membranes (0.2 µm) via Iblot 2 (7 min, 20 mV, Fisher Scientific). Membranes were then washed in TBST, blocked in 5% milk powder (MP) containing TBST for 1 h at RT on a rolling mixer, before being incubated in primary antibody (rabbit anti-γH2AX, Cat#9718, Cell Signalling, 1:1000) containing 5% bovine serum albumin (BSA, Sigma) TBST, supplemented with 0.05% sodium azide, overnight at 4^O^C. Secondary antibody conjugation was performed with goat anti rabbit-HRP (1:5000, Sigma) in 5% MP containing TBST for 1h at RT. Blots were washed in 3 × TBST for 5 min in-between each incubation step. Immunoreactivity was visualised with enhanced chemiluminescence (1.25 mM Luminol, 30 µM Coumaric acid and 0.015% H_2_O_2_) and captured with a Fuji LAS 4000 and imaging software (Fuji LAS image, Raytek). Following image capture, membranes were re-stained for Histone H3 loading control (Mouse anti Histone H3, Cat# 3638S, Cell Signalling, 1:5000) and secondary antibody goat anti-mouse-HRP (1:5000, Sigma) as described above.

Immunoreactivity was measured using ImageJ with area under curve function. γH2AX signals were normalised to Histone H3 loading controls. Quantification of control and DLB nuclear isolates were performed across multiple western blots and as such values were normalised within blots to control values prior to being pooled and probed for significance via a Mann–Whitney non-parametric t-test, with *p* < 0.05, being taken as significant.

### Mass spectrometry analyses of fractionated tissue

#### Protein digestion and quantitative proteomics

Protein digestion and proteomics were conducted in 3 separate batches each containing Con and DLB cases (*n* = 3 for each), with slight differences in sample preparation between batches (See Supplementary methods for details). For all runs, frozen temporal cortex samples (250 mg) were fractionated as above, nuclear pellets were resuspended, heated to 95°C and sonicated to clear DNA/RNA. Samples were then reduced, cystines alkylated and acidified with phosphoric acid (final concentration 2.5%). Protein digestion was carried out with S-Trap micro spin columns (Protifi). The samples were loaded onto spin columns in 6 volumes of binding buffer (90% methanol 100 mM TEAB pH 7.55) and centrifuged at 4000xg for 30 s. The columns were then washed with binding buffer (three times) and the flow through discarded. Proteins were digested with trypsin (Worthington) in 50 mM TEAB pH 8.5 (see supplementary for details). Peptides were eluted with three washes of the trap; first 50 µl 50 mM TEAB, second 50 µl 0.2% formic acid and third 50 µl 50% acetonitrile with 0.2% formic acid. The solution was frozen then dried in a centrifugal concentrator and reconstituted in 0.2% formic acid to a concentration of 0.5 or 1 µg/ml (depending on batch, see Supplemental methods).

Equivalents of 0.5 or 1 µg of each peptide sample (depending on batch, see Supplemental methods) were loaded per LCMS run (using an UltiMate 3000 RSLCnano HPLC), first onto a 300 μm × 5 mm C18 PepMap C18 trap cartridge (Thermo Fisher Scientific) in 0.1% formic acid at 10 µl/min for 5 min and then separated on a 75 μm × 50 cm C18 column (Thermo EasySpray -C18 2 µm) with integrated emitter, using a 110 min gradient from 97% A (0.1% FA in 3% DMSO) and 3% B (0.1% FA in 80% ACN 3% DMSO), to 35% B, at a flow rate of 250 nl/min. The separated peptides were then injected into Exploris 480 Quadrupole-Orbitrap Mass Spectrometer (Thermo Fisher Scientific, Waltham, MA, U.S.A.) via EasySpray source at the Ion Transfer Tube temperature of 280°C, spray voltage 1500 V and analysed using data dependent (DDA) acquisition. The total LCMS run time was 150 min. Orbitrap full scan resolution was 120,000, RF lens 50%, ACG Target set to “Standard”, Scan Range 400–1600 m/z. Top 20 method was implemented to select precursors for MSMS. MIPS set to peptide, Intensity threshold 5.0 e3, charge state 2–7 and dynamic exclusion after 1 time for 35 s 10ppm mass tolerance. ddMS2 scans were performed at 15,000 resolution, HCD collision energy 30%, first mass 110 m/z, ACG Target set to 100%.

### Bioinformatic proteomic analysis

The acquired data was searched against the human protein sequence database, available from (https://www.uniprot.org/uniprot/?query=proteome:UP000005640), concatenated to the Common Repository for Adventitious Proteins v.2012.01.01 (cRAP, ftp://ftp.thegpm.org/fasta/cRAP), using MaxQuant v1.6.43. Fractions were assigned as appropriate. Parameters used: cysteine alkylation: iodoacetamide, digestion enzyme: trypsin, Parent Mass Error of 5ppm, fragment mass error of 10 ppm. The confidence cut-off representative to FDR < 0.01 was applied to the search result file. Data processing was performed in Perseus [45]. All common contaminants, reversed database hits, and proteins quantified with less than 2 unique peptides were removed from the dataset. For quantitative proteomics, per case, intensity values for proteins were transformed to log2 and technical replicates averaged. Median was then subtracted within each sample to account for unequal loading and the width of the distribution adjusted. The dataset was filtered, keeping proteins with > 2 valid values in at least one experimental group. Furthermore, any protein which was not detected in 70% of all samples were removed. Remaining missing values were imputed from the left tail of the normal distribution (2StDev away from the mean, ± 0.3 StDev). Compositional values were used to determine any changes in the abundance of identified proteins between Con and DLB nuclear samples.

Given that proteomic analysis was conducted in 3 batches (containing 3 control and 3 DLB cases, each). Values were transformed into Z-scores per protein per batch to allow for disease comparison, negating the impact of different runs across samples.

### Determination of altered expression and functional enrichment

Comparisons of nuclear protein abundance between disease groups were performed using Students T-test (S_0_ = 0.1, with permutation-based FDR < 0.1, with 250 randomizations). Heatmaps of Z-scores per protein per case were plotted in Perseus. All proteins established as upregulated as per FDR < 0.1, were subject to STRING database [46] and DAVID Bioinformatics [47, 48] analysis. For establishing enrichment of functional terms, upregulated proteins (89 proteins) were compared against a reference background dataset of all detected proteins (1719) in DAVID. Go terms (GOTERM_BP_Direct, GOTERM_CC_Direct and GOTERM_MF_Direct) and Functional annotations (UP_KW_BIOLOGICAL_PROCESS, UP_KW_CELLULAR_COMPONENT and UP_KW_MOLECULAR_FUNCTION) were used for assessment of enrichment as per the default settings of DAVID bioinformatics. Output clusters and enrichment data were inputted into R, generating graphs reporting -Log10 FDR and enrichment per GO term and/or keyword. Further determination of specific DNA damage/repair processes involving the identified upregulated proteins was perform via STRING. The STRING database plots both physical and functional connections between proteins, compiling interaction evidence from multiple sources of available data [46, 49]. Sources include repositories of experimental biochemical data, gene coexpression and interactions reported in additional databases such as reactome (as per www.reactome.org) and Gene Ontology consortium (as per www.geneontology.org). Interaction evidence is then benchmarked against KEGG pathway maps and a confidence score (0–1) is generated. Here interaction maps are shown with a minimum confidence score of 0.4. Additional protein enrichment was also carried out on STRING database platform employing the Gene ontology consortium database. For this analysis the reference set used was the human genome and not the background dataset, as this analysis was intended purely to highlight specific functional pathways which may be affected from changes in the nuclear proteome.

## Results

### Nuclear aSyn from DLB tissue is seed-competent

**Fig. 1:**
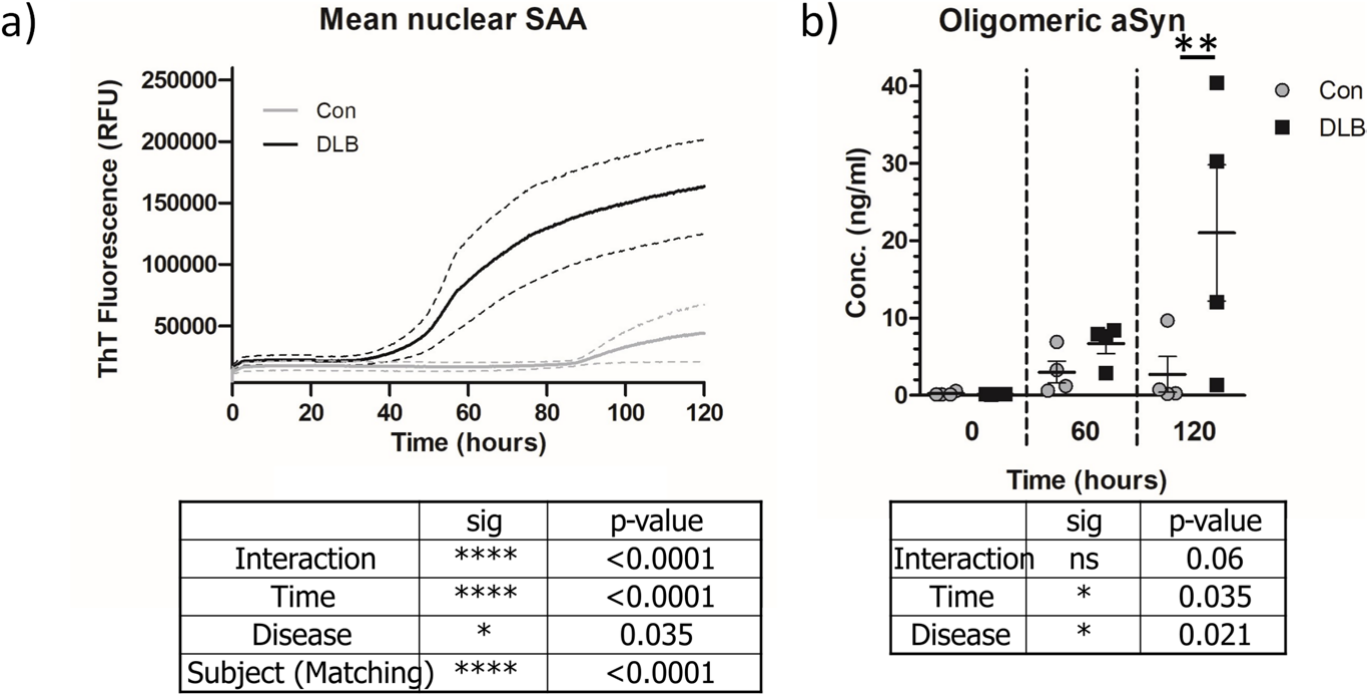
DLB nuclear extracts are seed competent. **a** Thioflavin T fluorescence traces from seed amplification assay (SAA) of nuclear extractions from controls (Con) and dementia with Lewy body (DLB) cases (*n* = 4, per group). Mean trace per group ± SEM (dotted lines) is shown. **b** SAA products taken at 20, 60 120h of reaction time were quantified for oligomeric aSyn concentration as detected via Syn-O2 antibody ELISA. Data shown as scatter plot of aSyn oligomer concentration per disease group at relevant time points. Data shown as Mean ± SEM. Outcome of 2 way ANOVA shown in corresponding plots and significant Bonferroni post-test indicated. * = *p* < 0.05, ** = *p* < 0.01, **** = *p* < 0.0001

Building upon our prior observations of increased S129 phosphorylation of nuclear aSyn and the presence of higher molecular aSyn species in DLB nuclei [28], we further probed the pathogenic properties of nuclear aSyn. Using an aSyn seeding amplification assay (SAA), the aggregation properties of nuclear associated aSyn, were examined. We observed a significant increase in aSyn aggregation in nuclear extracts from DLB cases vs. controls (Fig. 1a; 2-way repeated measures ANOVA, Time: F(478, 8) = 11.46, *p* < 0.001, Disease: F(1,8) = 7.34, *p* = 0.035 and Interaction: F(48,8) = 7.44, *p* < 0.001). No such aggregation was evident in all but one of the control cases (see Supplementary Fig. 1). Timepoint analysis of SAA products confirmed the elevated presence of oligomeric aSyn species in DLB nuclear extracts (Fig. 1b; 2-way ANOVA Time: F(2, 8) = 4.08, *p* = 0.021, Disease: F(1,8) = 5.51, *p* = 0.035).

### Elevation of double strand breaks in cortical tissue from DLB cases and from A30P aSyn mice

**Fig. 2:**
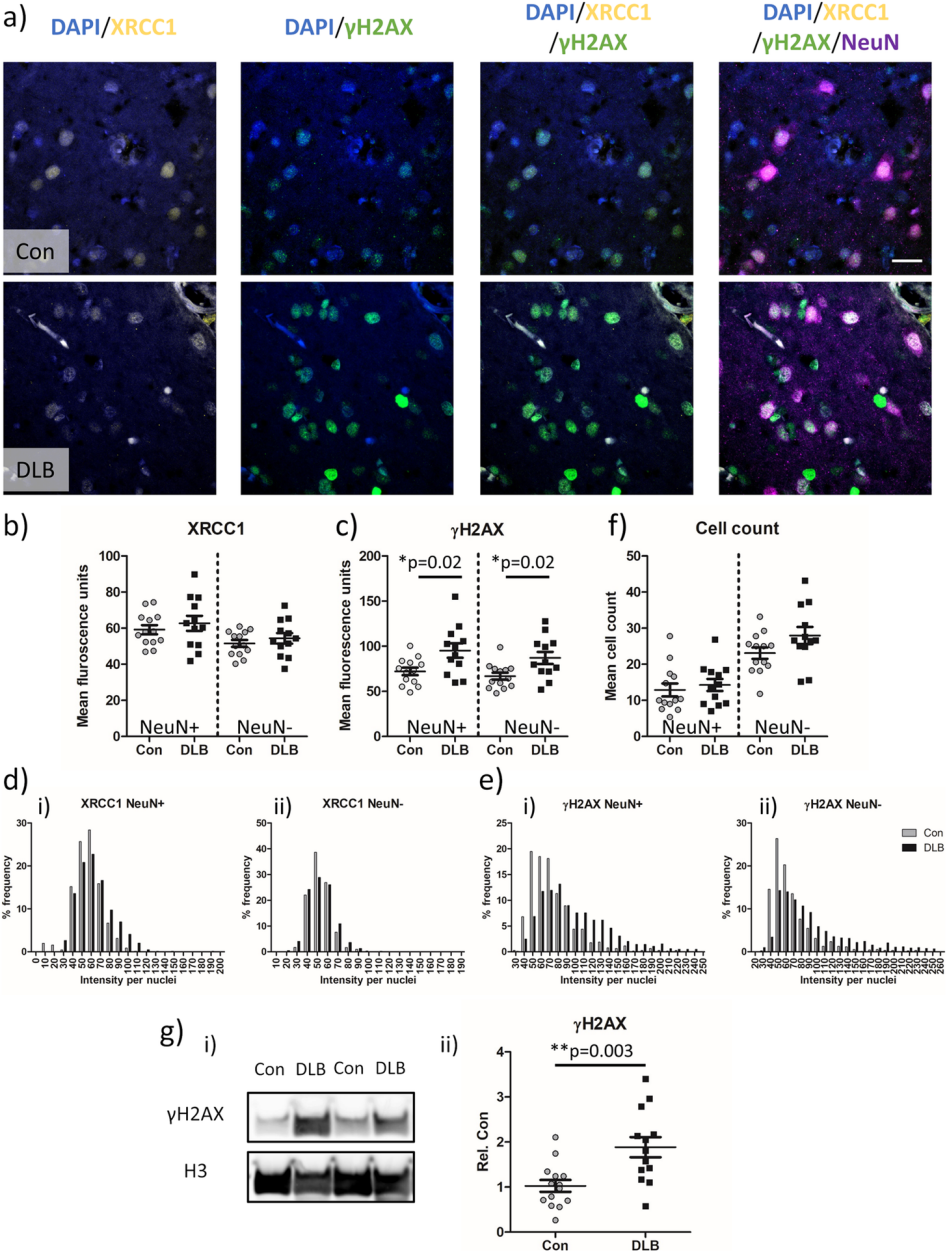
Double strand breaks are increased in temporal cortex nuclei of DLB cases. **a** Example confocal images (63 × objective) from control (Con) and cases of dementia with Lewy bodies (DLB) showing single strand break (XRCC1) and double strand break (γH2AX) associated immuno-fluorescence in neurons (NeuN) and non-neuronal cells. Nuclei are co-stained with DAPI. Quantification of mean **b**) XRCC1 **c**) γH2AX fluorescence signal, alongside **f**) nuclei based cell counts from Con (*n* = 13 cases) and DLB (*n* = 12 cases) are presented. Additionally, **d**i-ii) frequency distribution of XRCC1 and **e**i-ii) γH2AX per nuclei are shown. Each measure output is reported for neuronal (NeuN +) and non-neuronal (NeuN-) populations. **g**i Western blot images of γH2AX and loading control Histone H3 and **g**ii) quantification of loading adjusted γH2AX signal in Con and DLB cases (*n* = 14 and 13 respectively; g.ii) are also shown. Data are expressed in scatterplots with mean ± SEM (in b,c,f and g) and as percentage frequency of immunofluorescence intensity per bins of 10 units width (in d + e). Statistical outcome of Mann–Whitney non-parametric tests shown in appropriate plots, * = *p* < 0.05 and ** = *p* < 0.01. Scale = 20 µm

Given recent reports of a physiological role of aSyn in the regulation of DDR [15, 50], we next sought to establish how such pathological aSyn may impact on the genomic stability of cortical nuclei in cases of DLB. DNA damage markers of SSBs and DSBs were measured as per XRCC1 and γH2AX respectively, in NeuN-positive ( +) neurons and NeuN-negative (-) non-neuronal cells within the lateral temporal cortex of control and DLB cases (Fig. 2a). As expected immunoreactive signals of XRCC1 and γH2AX were concentrated within nuclei. Mean levels of SSBs in both NeuN + and NeuN- nuclei were comparable between control and DLB cases (*p* > 0.05, Fig. 2b). However, mean γH2AX levels were elevated in NeuN + and NeuN- nuclei in DLB cases compared to controls (*p* = 0.02 for both, *n* = 13 for control and *n* = 12 for DLB, Fig. 2c). Analysis of the frequency distribution of XRCC1 (Fig. 2d) and γH2AX (Fig. 2e) intensities in all nuclei measured, demonstrated a γH2AX selective, decrease in the frequency of peak intensities and a rightward extension of the intensity profile for both NeuN + and NeuN- nuclei in DLB cases compared to controls. Such a change in the distribution profile of γH2AX in DLB is consistent with an increase in nuclear DSBs in a large sub-population of cells. There was no significant difference in the mean number of NeuN + and NeuN-ve cells sampled per field between controls and DLB cases (Fig. 2f; *p* > 0.05).

For all cases, where available (*n* = 13 for controls and *n* = 11 for DLB cases), TMA slides containing temporal cortex tissue punches were stained for hallmark pathology associated with DLB, foremost Lewy neurite and Lewy body pathology, but also Aβ-plaques and AT-8 phospho-tau (Supplementary Fig. 2a). Analysis revealed a significant elevation of Lewy pathology (*p* = 0.0002), as well as amyloid plaques (*p* = 0.02) but not AT-8 reactive phospho-tau in DLB cases compared to controls (Supplementary Fig. 2b). Correlation between hallmark pathology burden and yH2AX, either as the complete cohort (Con + DLB), or when considered based on disease (Con or DLB), reported no significant relationship between plaque, tangle or Lewy pathology and DNA damage markers, in neuronal populations (Supplementary Fig. 2C). For non-neuronal cells, surprisingly a strong inverse correlation was observed with Lewy pathology and yH2AX (*p* = 0.02, r = −0.7) when considering the DLB cohort only (Supplementary Fig. 2c + d), potentially suggesting those case with more aggregated and less dispersible pools of aSyn have lower levels of glial based DNA damage. No other significant correlations were observed in the non-neuronal population. Moreover, no correlations were observed when considering neuropathological staging scores, although a DLB based group effect was observed between yH2AX and Braak LB stage, in both the neuronal and non-neuronal population (Supplementary Fig. 2e).

The elevation of nuclear γH2AX signal in DLB cases compared to controls was confirmed via western blot of isolated nuclear lysates from frozen lateral temporal cortex (Fig. 2g, *p* < 0.003, *n* = 14 for controls and *n* = 13 for DLB, see Supplementary Fig. 3 for full blots).

**Fig. 3:**
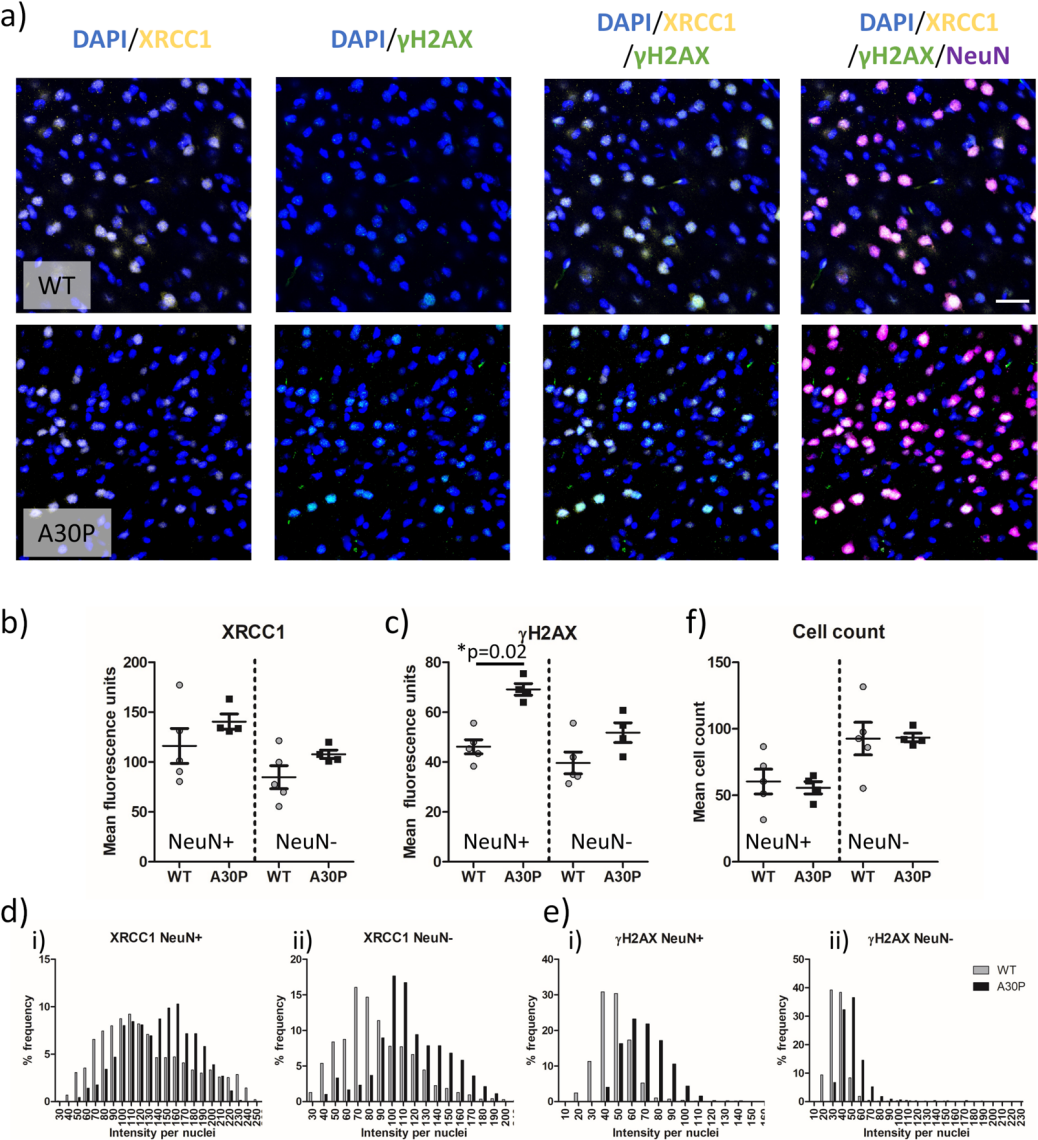
Double strand breaks are increased at 3 months of age in an A30P mouse model of synucleinopathy. **a** Immunofluorescence confocal images (63 × objective lens) of XRCC1, γH2AX, NeuN and nuclei co-stained with DAPI from the somatosensory cortex of wild type (WT) and A30P mice. Quantification of neuronal (NeuN +) and non-neuronal (NeuN -) mean **b**) XRCC1 and **c**) γH2AX signal in WT (*n* = 5) and A30P mice (*n* = 4). Frequency distribution of **d**) XRCC1 and **e**) γH2AX per nuclei are shown in NeuN + (i) and NeuN – (ii) nuclei are also shown alongside **f**) cell count, as per nuclei number. Data are expressed in scatterplots with mean ± SEM (in b, c and f) and as percentage of immunofluorescence intensity binned with 10 unit widths (d and e). Statistical outcome of Mann–Whitney non-parametric tests shown in appropriate plots, * = *p* < 0.05. Scale = 20 µm

In the A30P mouse model, following the statistical identification of an outlier in the data as per Grubbs test (see Supplementary Fig. 4), similar changes were observed in the somatosensory cortex at 2.5–3 months of age (Fig. 3a-c). However, in accordance with the neuronal specific expression of human mutant aSyn in these mice, levels of the DSB marker γH2AX were only significantly elevated in the NeuN + neuronal population (*p* = 0.02, Fig. 3c) and not in non-neuronal nuclei (*p* > 0.05). Notably, despite no statistical significance, mean values of the SSB marker XRCC1 were increased in A30P mice compared to WT. The trend for an increase in XRCC1 in A30P mice was further supported by the apparent shift in the frequency distribution of XRCC1, suggesting a modest increase in SSBs in addition to increased DSBs. The expression of γH2AX in neuronal NeuN + and non-neuronal NeuN- nuclei (Fig. 3d + e) also demonstrated a pronounced shift in the distribution of intensities. The rightward shift was indicative of an increased percentage of nuclei with high levels of both SSBs and DSBs within the somatosensory cortex, as a consequence of the expression of mutant human aSyn in A30P mice. Despite the elevation of DNA damage in these young A30P mice there was no evidence of cell loss, as neuronal and non-neuronal numbers were comparable between WT and A30P mice (Fig. 3f, *p* > 0.05).

### Pathological nuclear aSyn correlates with an increase in DSBs

**Fig. 4:**
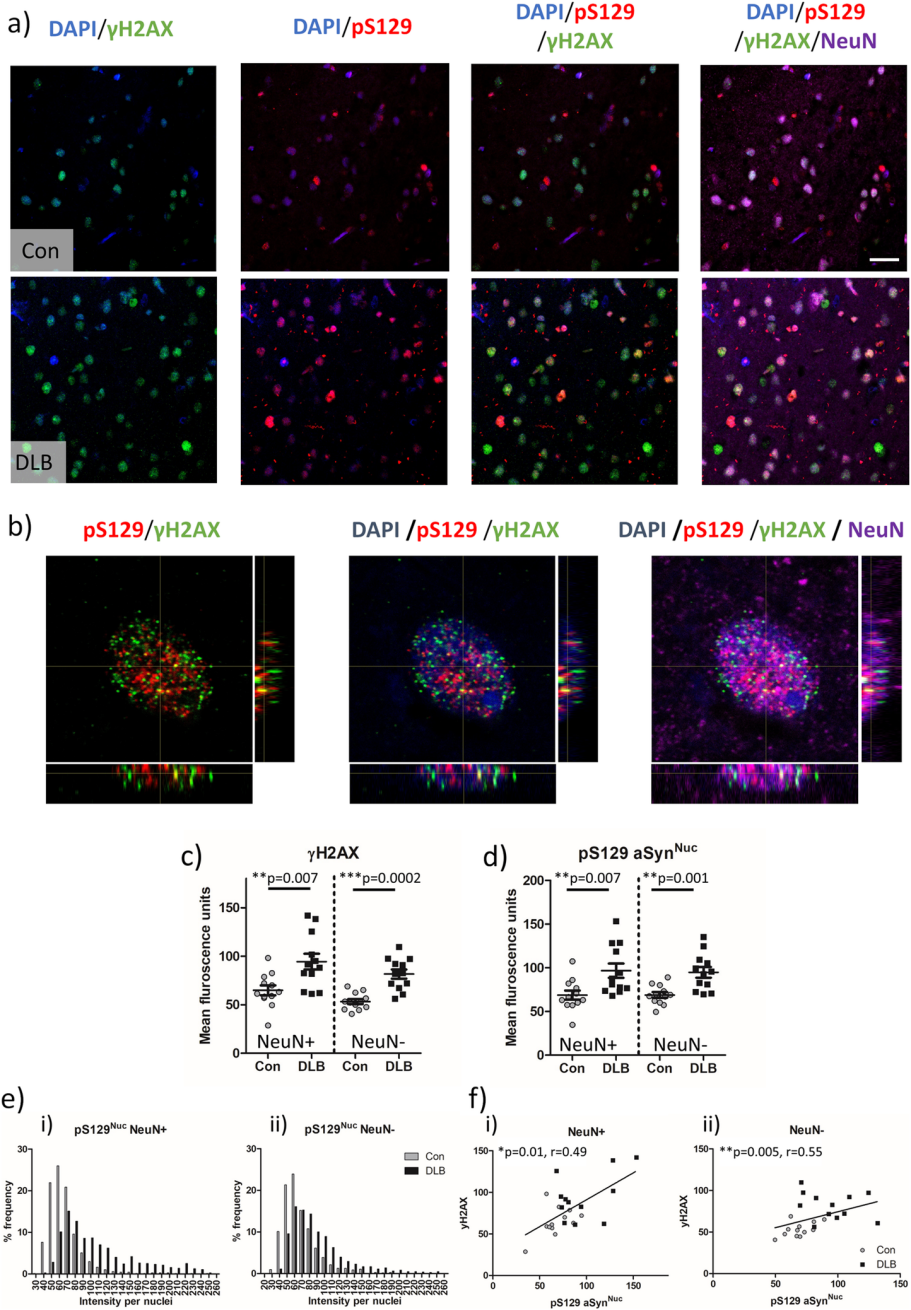
Phosphorylated aSyn increases in concert with double strand breaks and correlate with each other in the nuclear compartment. **a** Example confocal images (63 × objective) from control (Con) and cases of dementia with Lewy bodies (DLB) showing alpha-synuclein phosphorylated at serine 129 (pS129), double strand break (γH2AX) immuno-fluorescence in neurons (NeuN) and non-neuronal cells.. Intra-nuclear yH2AX and ps129 signal were confirmed via **b**) Z-stack reconstructed orthogonal views of digitally zoomed neuronal nuclei images captures from DLB cases. Quantification of **c**) nuclear pS129 (pS129 aSyn^Nuc^) and **d**) γH2AX fluorescence from Con (*n* = 13 cases) and DLB (*n* = 12 cases) are presented. **e**i-ii Frequency distribution of ps129 (per nuclei) are shown as well as **f**i-ii) correlative levels of ps129 aSyn^Nuc^ with levels of γH2AX, per case, with spearman's correlation (r) reported. Measures are reported for neuronal (NeuN +) and non-neuronal (NeuN-) populations. Data are expressed in scatterplots with mean ± SEM (in c, d) and as percentage frequency of immunofluorescence intensity per bins of 10 units width (in e). Statistical outcome of Mann–Whitney non-parametric tests and Spearman’s (r) correlation shown in appropriate plots, * = *p* < 0.05, ** = *p* < 0.01 and ** = *p* < 0.001. Scale = 20 µm

To determine whether nuclear aSyn pathology correlated with the observed increase in DNA damage, we quantified the levels of phosphorylated nuclear aSyn (pS129) and of DSBs (γH2AX) using immunostaining in control and DLB human tissue (Fig. 4). Consistent with the initial observations (Fig. 2), DSBs were observed as increased in both NeuN + and NeuN- nuclear populations of DLB cortical tissue compared to controls (Fig. 4a + c). In line with the relative infrequent occurrence of cytoplasmic aSyn aggregation even in DLB cases, the majority of pS129-aSyn immunoreactivity was seen in the form of Lewy neurites, although also evident as previously reported [28], was nuclear pS129 aSyn in both control and DLB tissue (Fig. 4a). Higher magnification Z-stack images of DLB nuclei and orthogonal reconstruction supports the intranuclear localisation of both yH2AX and pS129 aSyn (Fig. 4b). Intra-nuclear ps129 aSyn was also increased in the DLB cohort, in NeuN + and NeuN- nuclei (*p* = 0.007 and *p* = 0.001 for NeuN + and NeuN- respectively, Fig. 4a + d). Similarly, a rightward shift in the distribution profile of mean intensity of nuclear pS129 aSyn was seen in NeuN + and NeuN-ve nuclei populations (Fig. 4e.i + ii), indicative of a rise in nuclear pS129aSyn in a subpopulation of cells. Correlative analysis per case of the mean intensity of γH2AX and nuclear pS129 aSyn, reported significant linear relationships in both NeuN + (Fig. 4f.i, r = 0.49, *p* = 0.01) and NeuN- (Fig. 4f.ii, r = 0.55, *p* < 0.005) nuclei, supportive of a potential interaction between nuclear aSyn pathology and elevated DSBs.

### LBs contain genomic chromatin originating from DSBs

**Fig. 5:**
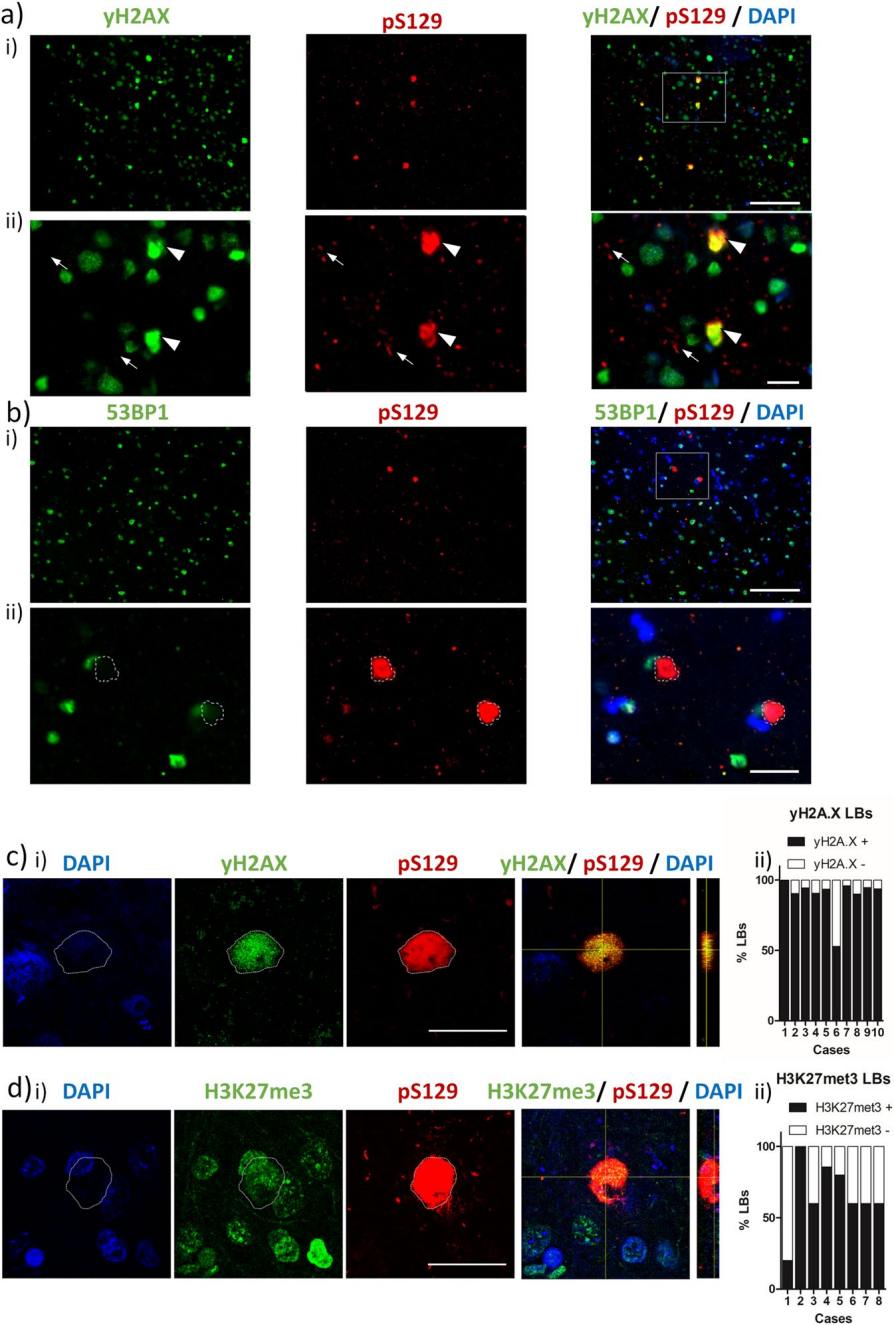
Nuclear material is a core constituent of cortical Lewy bodies. **a** Example widefield images (i. 20 × objective) cases of dementia with Lewy bodies (DLB) showing Lewy bodies (LBs) as detected via alpha-synuclein phosphorylated at serine 129 (pS129), $\gamma$ H2AX and **b**) 53BP-1 immuno-fluorescence, with nuclei are co-stained with DAPI. Digitally zoomed images (ii) show LBs immunoreactivity (arrowhead) and Lewy neurites (arrows) in greater magnification. Note the colocalization of γH2AX with extranuclear pS129 LBs (a) and absence of colocalization of 53BP-1 with LBs (b). **c** Confocal image of YH2AX and **d**) H3K27me3 reactive LBs. Quantification of **c**ii) γH2AX and **d**ii) H3K27me3 positive (γH2AX +) and negative (γH2AX -) LBs in examined cases. Data shown as percentage LBs positive and negative for γH2AX and H3K27me3. Scale bar in a.i + b.i = 100 µm, in a.ii + bii = 50 µm and in c + d = 20 µm

Remarkably, the DSB marker γH2AX was not only present in the nuclei of cortical cells in fixed tissue sections but was also apparent in pS129-aSyn rich LBs within DLB cases (Fig. 5a). In comparison to the robustly labelled γH2AX LBs (white arrow heads, Fig. 5a.ii), Lewy neurites (white arrows) were negative for the DSB marker. Despite the prominence of this nuclear DSB marker, LBs were negative for a secondary nuclear marker 53BP-1 (Fig. 5b.i, dotted LB outline in Fig. 5b.ii). Such an immunoreactivity profile is consistent with the genomic chromatin originating from cytoplasmic chromatin fragments (CCFs), downstream from excessive unrepaired DNA damage [8]. γH2AX immunoreactivity within LBs was confirmed via confocal microscopy, such that γH2AX puncta were evident throughout the Lewy body structure (Fig. 5c.i, for additional examples see Supplementary Fig. 5). Across 10 DLB cases, a remarkably high percentage of LBs were positive for γH2AX (Fig. 5c.ii, mean 89.6 ± 4.2%). Such frequency of γH2AX immunoreactivity was largely consistent across cases (9 of 10 cases, with 90–100% of LBs positive for γH2AX, with only one case of lower frequency of 52.9%), suggesting that the inclusion of DSB originating genomic material within LBs is a common occurrence. Moreover, this is consistent with the possibility of the entrapment of damage generated nuclear DNA within LBs. Accordingly, a secondary marker of nuclear DNA damage and specifically CCFs, H3K27me3 [8, 51], was also positive in the majority of LBs examined across 8 cases (Fig. 5d, for additional examples see Supplementary Fig. 6). Though the inclusion of H3K27me3 in LBs was not as frequently detected as yH2AX.

### DNA damage repair is upregulated in DLB cortical tissue

Given the widespread elevation of DSBs within DLB cortical tissue, and the potential involvement of DSB related genomic material in the cytoplasmic aggregation of aSyn, a key aspect to the cellular pathology underlying DLB is likely to be disease-dependent changes in the nuclear proteome.

**Fig. 6:**
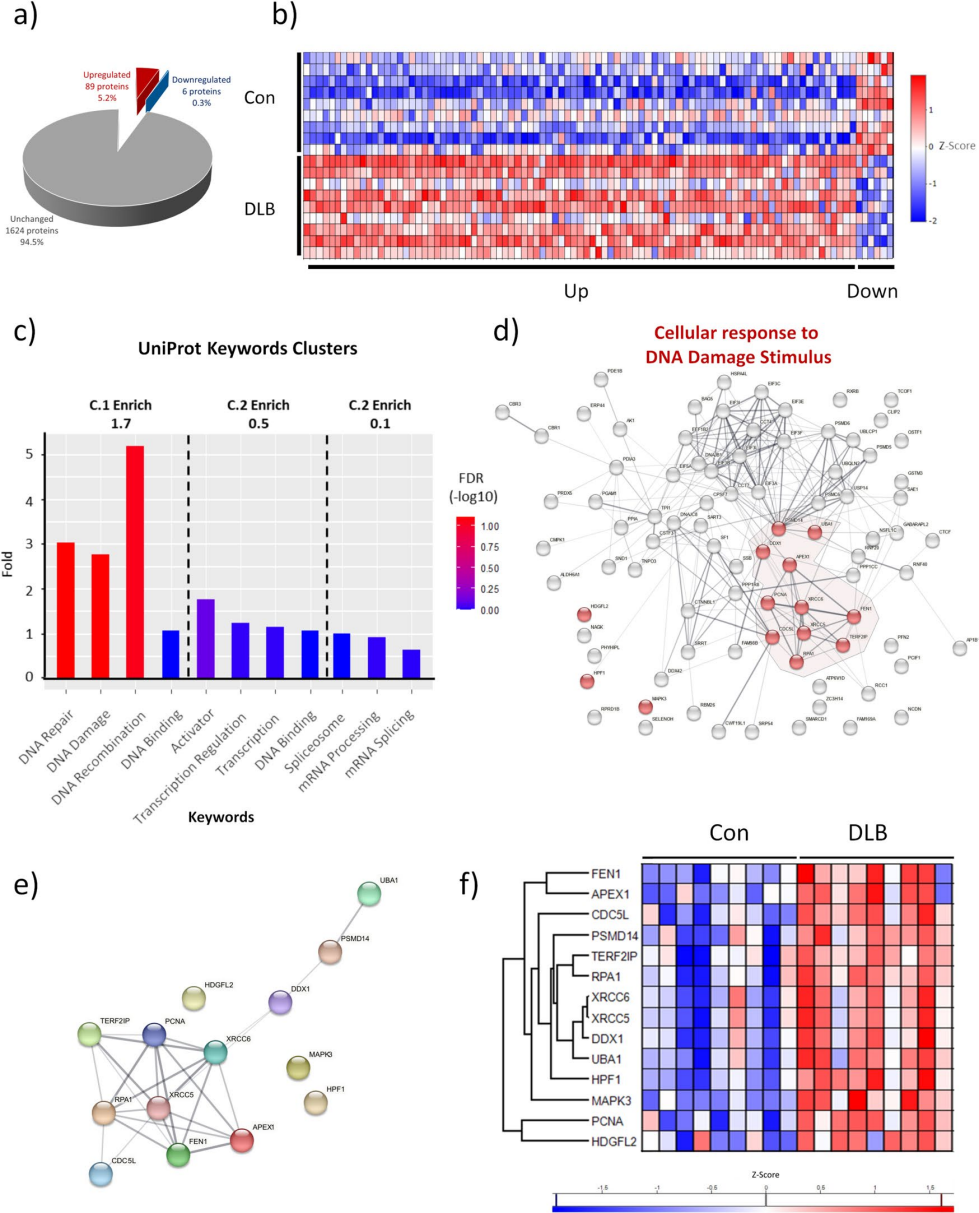
Altered nuclear proteome of DLB temporal cortex tissue. **a** Upregulated, downregulated and unchanged nuclear proteins in temporal cortex of dementia with Lewy body cases (DLB), compared to controls (Con). Data provided as absolute numbers and percentages of total identified proteins. **b** Z-score heatmap of altered proteins between Con and DLBs. **c** Uniprot keyword enrichment of the 89 proteins established as elevated in nuclear proteome of DLB compared to controls. Fold enrichment and significance is shown as per false discovery rate for each individual keyword. Keywords are grouped into clusters as reported per the DAVID bioinformatic database. Dotted lines denote the boundaries of clusters, with cluster enrichment (C.Enrich) from background reference database shown within each cluster. **d** STRING map of protein interactome of upregulated proteins. Prominent functional category of “Cellular response to DNA damaging stimulus” is highlighted (red) with individual proteins associated with each term denoted by colour and hub of strong interaction associated highlighted by background colour. Physical and functional interactions are shown with the weighting of line indicating confidence in interactions. **e** STRING interaction map and **f**) Z-score heat map of upregulated proteins associated with “Cellular response to DNA damaging stimulus” plotted in isolation

Accordingly, proteomic analysis of nuclei, isolated from the lateral temporal cortex of DLB and control tissue (*n* = 9 for each) was conducted. Of the reported 1719 identified proteins detected consistently across conditions, 89 (5.2%) were upregulated and 6 (0.3%) were downregulated in DLB tissue (Fig. 6a + b, for full data set see Supplementary Table 2 and for protein labelled heat map see Supplementary Fig. 7). No protein was reliably detected only in either control or DLB cases.

Functional enrichment cluster analysis of upregulated proteins from background (1719 detected proteins) against Uniprot keywords as per DAVID bioinformatics, revealed a primary enrichment cluster (1.7-fold enriched) of DNA damage, repair, recombination and binding. Minimally enriched secondary (0.5-fold enriched) and tertiary (0.1-fold enriched) clusters associated with transcription and mRNA processing and splicing (Fig. 6c** + **Supplementary Table 3), were also identified. Similarly, analysis of upregulated proteins for enriched GO terms reported a cluster (1.7-fold enriched) associated with Damaged DNA binding, DNA recombination and Telomeric DNA maintenance and a secondary cluster (1.2 fold enriched) associated with DNA Repair, base-excision repair and binding of damaged DNA. Nevertheless, in relation to the GO terms, no individual terms were identified as significantly enriched following adjustments for multiple comparisons (See Supplementary Fig. 8 and Table 4). Moreover, the major enriched GO term cluster (3.2-fold enriched) was associated with the eukaryotic translation initiation factor 3 complex (EIF3), typically associated with protein translation. However, EIF3 family members are detected in the nuclear compartment, complexed with histones [52], and EIF3E has known roles in DDR [53]. Likewise, a cluster associated with the ubiquitin proteasome system (UPS), which is associated with a variety of processes required for DDR, was also identified (1.2-fold enriched) [54–56]. Interestingly, whilst our proteomics did detect RNA binding proteins NONO and SFPQ, which have recently been demonstrated to form nuclear aggregates in DLB brain tissue [57], we did not observe a significant increase in the abundance of these proteins, presumably due to the lack of comparison between soluble and insoluble fractions in our analysis.

To further understand the interactions of the upregulated proteins, additional STRING database analysis was conducted (Fig. 6d). From the 89 upregulated proteins, 87 were mapped and reported a strong level of interaction (87 nodes, 249 edges, average node degree 5.72, PPI enrichment *p* < 1.0e^−16^). Visual inspection of the protein interaction network demonstrated a prominent biological process cluster associated with “Cellular response to DNA damaging stimulus” (GO:0006974; Fig. 6d). Additionally major members of the “Ribonucloprotien complex assembly” (GO:0022618) and “Cellular macromolecule catabolic process” (GO:0044265) in relation to UPS were also evident (see Supplementary Fig. 9). Such terms were, however, only evident when upregulated proteins were compared to the whole human genome (Supplementary Table 4), as no significant enrichment was reported when compared to the nuclear proteome dataset (1719 detected proteins).

Regardless, when upregulated proteins within the “Cellular response to DNA damaging stimulus” were analysed in isolation (Fig. 6e + f), a strong protein–protein interaction was reported (14 nodes, 26 edges, average node degree 3.71, PPI enrichment *p* = 5.193e^−09^). Key biological functions, including “Base-excision Repair” and “Double strand break repair via non-homologous end-joining”, were identified within the top 10 (see Table 2 for top 10 terms and Supplementary Table 5 for full list).

**Table 2:** Go terms associated with enriched protein groups. Top 10 GO terms, based on enrichment strength when upregulated list is limited to those proteins associated with the cellular response to DNA damage. Provided are the GO term identification number (Go Term ID), the number proteins observed (observed) from the background protein set (background), the strength of the enrichment (strength), the false discover rate (FDR) and protein names (Proteins) of those identified

GO Term ID	Term description	observed	background	strength	FDR	Proteins
GO:0032201	Telomere maintenance via semi-conservative replication	2	7	2.6	0.0073	FEN1,PCNA
GO:0006287	Base-excision repair, gap-filling	3	14	2.48	0.00014	APEX1,FEN1,PCNA
GO:0071481	Cellular response to X-ray	2	12	2.37	0.0145	XRCC6,XRCC5
GO:0043247	Telomere maintenance in response to DNA damage	2	14	2.3	0.0183	APEX1,TERF2IP
GO:0070198	Protein localization to chromosome, telomeric region	2	17	2.22	0.0231	TERF2IP,XRCC5
GO:0006284	Base-excision repair	4	43	2.12	2.43E-05	APEX1,RPA1,FEN1,PCNA
GO:0007004	Telomere maintenance via telomerase	2	22	2.11	0.0353	RPA1,TERF2IP
GO:0000723	Telomere maintenance	7	97	2.01	9.68E-10	APEX1,RPA1,TERF2IP,FEN1,XRCC6,PCNA,XRCC5
GO:0006303	Double-strand break repair via nonhomologous endjoining	3	43	1.99	0.002	XRCC6,XRCC5,PSMD14
GO:0032508	DNA duplex unwinding	4	89	1.8	0.00028	RPA1,XRCC6,DDX1,XRCC5

Interestingly, several proteins associated with DDR were also present within the upregulated proteins, including the aforementioned EIF3E protein and key members of the UPS, but also many additional modulators of various DNA repair processes (See Supplementary Table 6).

## Discussion

In the present study, we report widespread DSB accumulation in the lateral temporal cortex of DLB cases, as well as in pre-symptomatic A30P human aSyn expressing mice. DSBs coincided with increased phosphorylated nuclear aSyn and a seed competent nuclear environment in DLB cases. Strikingly, the DSB marker γH2AX was also observed within LBs as was a secondary marker H3K27me3, suggestive of a reciprocal relationship between aSyn pathology and genomic damage. Accordingly, we observed a robust upregulation of DNA damage and repair proteins, as well as proteins associated with additional processes such as UPS and histone associated ribonucleoproteins within the nuclear proteome of DLB temporal cortex. Collectively the data suggest an association of nuclear aSyn pathology with genomic DNA damage, which in turn is associated with cytoplasmic aSyn pathology.

### Nuclear seed amplification and aSyn pathology

Building on our prior studies of the nuclear localisation [20, 28, 58], and pathological modification of aSyn in DLB cortical tissue [28], we now demonstrate the presence of pathological seed-competent aSyn in DLB nuclear extracts. Although it is well established that extracts from synucleinopathy brain tissue and recombinant aSyn fibrils are capable of seeding aggregate amplification [42, 59–61], we now demonstrate, the existence of seed-competent aSyn species in nuclear extracts, suggesting pathological alterations may take place in sub-cellular compartments with specific biochemical environments. It does however remain possible that trace cytoplasmic aSyn or aSyn associated with nuclear envelope [62], may be templating this aggregation. Though our prior characterisation of nuclear fractions [28] and our immunohistochemical data here would be consistent with the seeding of aggregation via the presence of intranuclear pool of pathological aSyn. Indeed, recent reports of nuclear aSyn in DLB cases with antibodies preferential binding to aSyn oligomers would appear to support the intranuclear localisation and aggregation of the protein, particularly co-aggregation with the RNA regulator protein SFPQ [57]. Regardless, given the pelleting of nuclei prior to homogenate use in standardised SAAs protocols, this nuclear seed represents a secondary aggregation source, in addition to the presumably cytoplasmic aSyn seeds within conventional homogenates. Whilst our data are highly supportive of the presence of endogenous pathological nuclear aSyn seeds, it remains possible that disease-dependent histone modification, also capable of inducing aSyn aggregation [9], may drive the observed nuclear seed amplification. Interestingly, this raises the possibility of histone driven aSyn aggregates exiting the nucleus and cross-seeding cytoplasmic aggregation. Regardless, the observed pathological aSyn seeding is consistent with our prior observations of increased aSyn phosphorylation and high molecular weight aSyn species in the nuclei of DLB cortical tissue [28].

### aSyn pathology and DNA damage

Disease-dependent modification of aSyn occurred alongside the accumulation of DSBs as per γH2AX in neuronal and non-neuronal cells in the temporal cortex of DLB cases. γH2AX results from the rapid serine 139 phosphorylation of histone H2AX, which is instrumental in initiating DSB repair, but is then dephosphorylated during the repair process or upon completion of repair [63, 64]. Accordingly, increased γH2AX is consistent with either an increased occurrence of DSB and/or a diminished capacity for DSB repair, and ultimately demonstrates a disruption of cellular genomic stability in DLB.

Similar reports of increased DSBs in PD cases [24, 65], demonstrate a robust link between aSyn pathology and DNA damage, irrespective of LBD subtype. However, unlike observed elevations of SSBs in PD brains [24], we did not detect an increase in SSBs in our DLB cohort based on XRCC1 immunoreactivity, (although see below for relevant discussion of A30P data). In the absence of an increase in SSBs, which can ultimately form DSBs [66], and in light of reports of decreased rates of DBS repair in *SNCA* knockout [15] and in A53T aSyn [16] expressing models, it is likely that the increase in DSBs we observed is a consequence of diminished DSB repair. DSB accumulation is observed in several model systems utilizing aSyn overexpression [20, 22], and pre-formed fibrils [67]. Thus, together these studies suggest that pathological aSyn accumulation, or modification, may mimic a loss of physiological function in DNA repair as seen in knockout and in mutant aSyn expressing models.

Indeed, it has previously been suggested that cytoplasmic aSyn aggregation may sequester nuclear aSyn from its repair function, with a modest association of higher DSB levels in aggregate containing neurons, supporting such a hypothesis [15]. However, our analysis of DSBs here, reveals an elevation across neuronal and non-neuronal cells, as well as a lack of correlation between DSBs and regional Lewy pathology within cases, thus such an elevation of DNA damage, appears to extend beyond LB burdened neurons. Likewise, elevated DSBs in A30P mice at a young age (3 months), precede robust cytoplasmic pS129aSyn accumulation and symptomatic presentation [38], yet is in line with the detection of nuclear ps129 aSyn at around 4 months of age [68]. Therefore, impaired DSB repair appears to occur early in the DLB pathological process, independently of the development of mature cytoplasmic aSyn aggregation.

Whilst some studies support DSB repair to be more strongly affected by aSyn pathology, our measures of SSBs in A30P mice confirm an appreciable increase in XRCC1 compared to controls, although non-significant. Given that unresolved SSB can generate DSBs [66], perhaps initial DDR dysfunctions may occur in SSB repair, later progressing to an accumulation of DSBs and a failure of their repair. It is equally plausible, however, that in addition to the failure of DSBs repair, early mitochondrial dysfunction and increased oxidative stress may independently drive an increase in SSBs [3]. Establishing a more detailed timeline of DNA damage in the context of organelle specific dysfunction will be an important focus of future research.

In A30P mice, the increased yH2AX and XRCC1 in the non-neuronal population, which do not express the Thy-1 driven SNCA transgene, although not significant, may imply a role for transcellular aSyn spread from neuronal aSyn release and glial uptake, foremost microglia and oligodendrocytes [69]. Glial aSyn uptake may also explain our observation of nuclear aSyn within non-neuronal cells in post-mortem human tissue and somewhat the inverse relationship between aggregated less dispersible Lewy pathology and elevated levels of yH2AX in the non-neuronal population of DLB cases. Such transcellular spread of aSyn is consistent with the development of cytoplasmic and nuclear inclusions within oligodendrocytes as part of MSA pathology [70], despite the lack of SNCA transcript within these cells [71].

In the present study aSyn pathology as per pS129-aSyn, was predominantly observed in the nucleus of cells and only occasionally within the perikaryal, in contrast to prior reports [40, 72, 73]. The reasons for this remain unclear, although it should be considered that the frequency of observing perikaryal based aSyn accumulation is dependent on the region examined. Perikaryal aSyn accumulations typically being more frequent in sub cortical and brain stem regions compared to cortical regions [74]. Moreover, the detection of different subcellular aSyn pools likely depends on the use of pan, oligomeric and/or post-translational modification specific antibodies [74, 75] as well as the antigen retrieval methods employed [28]. Beyond technical considerations, serine 129 phosphorylation may occur at least in the somatic cytoplasm after the deposition of aSyn into LBs, rather than prior to or during a state of diffuse aSyn accumulation [72]. As such detection of pathological perikaryal aSyn may be better detected via non-phosphorylation dependent antibodies. Moreover, whilst recent reports support a physiological role for pS129 aSyn at the synapse, where alterations in protein–protein interactions regulate synaptic vesicle recycling [76], such physiological and presumably modest phosphorylation may be obscured via passive post-mortem dephosphorylation in human brain samples akin to observations relating to the physiological phosphorylation of tau [77].

Nevertheless, here, the extent of DSBs within a given case correlated with nuclear pS129-aSyn levels, and only inversely correlated regional Lewy pathology burden in non-neuronal cells. Moreover, no correlations were observed with additional neuropathological hallmark pathology such as Aβ plaques and tau neurofibrillary tangles. This is surprising given the reports of elevated DSBs within AD cases [13], though an investigation of related Aβ and tau pathology proximal to sampled nuclei or indeed intranuclear Aβ and tau species remains to be conducted. This may be particularly pertinent for various tau species as growing evidence implicates nuclear tau dysfunction in neurodegenerative conditions and an association with altered DNA damage [78, 79]. Nevertheless, pS129-aSyn is a prominent pathological hallmark of synucleinopathies and is widespread throughout PD and DLB brain tissue [80]. Similarly, elevations of nuclear pS129-aSyn occur early within genetic aSyn mouse models, including in A30P mice, where nuclear pS129 aSyn is amongst the earliest pathological changes [68]. The coincidence of nuclear pS129 and DSBs in both human disease tissue and in mouse models is indicative of the potential for aSyn phosphorylation to alter its modulation of DDR. This is consistent with the differential DNA-binding behaviour of pS129 aSyn compared to non-phosphorylated aSyn [15, 50] as well as with the phospho-regulation of aSyn nuclear import [20, 58, 81]. Nevertheless, nuclear overexpression of aSyn can recapitulate PD related neurodegeneration and symptomatology in mice, independent of either mature aggregation or phosphorylation [82]. Thus, the specific disease related modification of aSyn which may drive the accumulation of DSBs in DLB remains unclear. It will also be important, in future studies, to assess the pathological properties of aSyn in MSA, a synucleinopathy characterized by the accumulation of aSyn protofilaments with a different folding structure and which demonstrate differential potency in cellular assays when compared to DLB /PD derived fibrils [83, 84].

### Presence of DSB in LBs

Strikingly, we found that the principal marker of DSBs, γH2AX was also evident within the majority of cortical LBs examined. Several studies have highlighted the presence of DNA within the core of LBs, either via extranuclear DAPI signal [73, 85] or more specifically through the use of dsDNA antibodies [86], although this has largely been assumed to be mitochondrial in origin. Here, the histone marker yH2AX as well as a secondary marker of DNA damage H3K27me3, raises the possibility that such DNA may also contain DNA of nuclear origin. It must nevertheless be acknowledged that at present colocalization of dsDNA and histone markers remains to be fully demonstrated. However, the occurrence of ectopic cytoplasmic genomic material, either micronuclei or the aforementioned CCFs are forms of cytoDNA, produced as a consequence of chronic unresolved DNA damage [8]. Interestingly CCFs are known to be positive for yH2AX and H3K27me3, as well as negative for 53BP1, supporting the potential entrapment of such cytoDNA species within LBS. A more detailed assessment of CCF and/or micronuclei markers will be required in the future. Nevertheless, given that double stranded DNA [10] and histones [9], the major components of chromatin, have pro-aggregation properties towards aSyn, it is possible that genomic DNA damage mediated cytoDNA may act as co- aggregates, facilitating the formation of cytoplasmic aSyn aggregates in-situ. Whether this is true for all LBs is unclear and given the structural differences between cortical and brain stem LBs [73], we are cautious to generalise, without further determining the inclusion of nuclear DNA damage markers in “classical” brain stem LBs.

### Alteration of nuclear DNA damage repair pathways

**Fig. 7:**
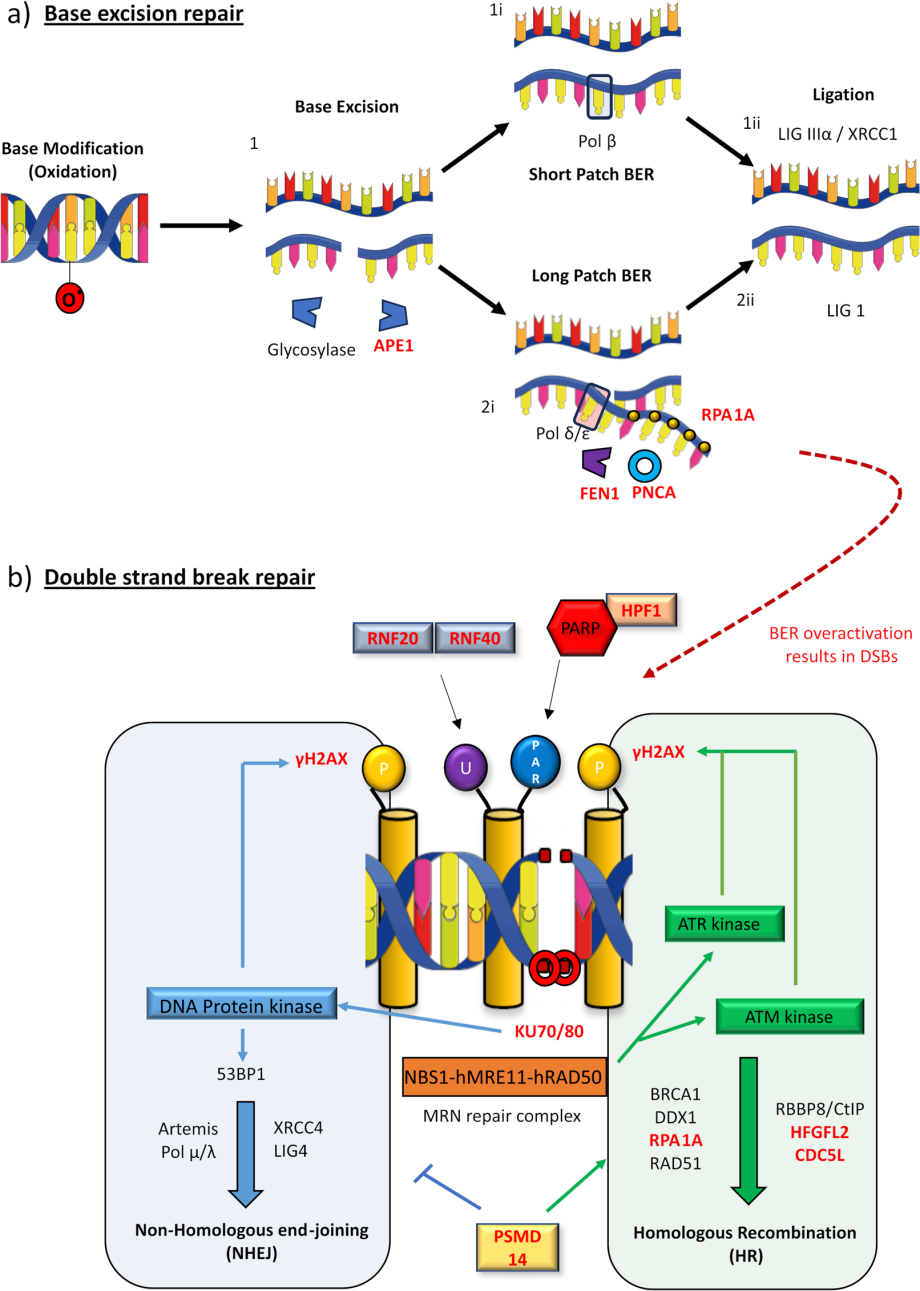
Upregulated protein components of DDR in DLB and principal pathways. **a** Base excision repair (BER) diagram. Following oxidation (as shown), alkylation or deamination, modified bases are excised via glycosylase activity and deoxyribose-phosphate DNA backbone cut by APEX1 [1]. Repair proceeds as short patch (i) or long patch repair (ii). In short patch BER, the single nucleotide gap is repaired by β-polymerase (2.i) and the DNA ligase IIIα-XRCC1 complex (2.ii). Long patch BER, activated in cases of excess oxidation, produces a 2–8 nucleotide sequence by δ/ε polymerases, which is bound to replication protein 1 A (RPA1A). Excessive sequence is then cleavage by FEN1, facilitated by proliferating nuclear protein A (PCNA) (2.ii), before ligated by DNA ligase I [99, 100] (3.ii)**.** When overactive, BER can result in double strand breaks (DSBs) [91–95]. **b** Non-homologous end-joining (NHEJ) and homologous recombination (HR) DSBs repair pathways. In NHEJ, DSBs are sensed by DNA–protein kinase (DNA-PK) regulatory subunits KU70/KU80, activating DNA-PK, enabling histone phosphorylation generating DSB signal γH2AX. Surrounding chromatin DSB is relaxed, in part due to histone ubiquitination (U) via ubiquitin ligases including ring finger 20/40 (RNF20/40) [56]. NHEJ components are recruited to the DSB, including the 53BP1, Artemis nuclease, DNA polymerase µ/λ and XRCC4/DNA ligase 4 [96]. HR is initiated via the DSB sensing of the MRN repair complex, activating Ataxia-telangiectasia mutated (ATM) and ataxia telangiectasia and Rad3-related (ATR) protein kinases, generating γH2AX signal [3]. CDC5L is a regulator of ATR kinases [101]. DSBs strands are resected by MRE11 promoted by BRCA1 and RBBP8/CtIP [3], facilitated by HDGFL2 [102], RNA–DNA hybrids are cleared via DEAD Box 1 (DDX1) [103, 104], prior to RP1A coverage, RAD51 guided homologous DNA search and strand invasions prior to holiday junction formation and repair [3]. HR engagement over NHEJ is part co-ordinated by PSMD14, which restricts the accumulation of NHEJ inducer 53BP1 at damaged sites [105]. DDR protein upregulated in DLB nuclei highlighted in red

Concordant with widespread DSB accumulation, nuclear proteomic analysis highlighted an upregulation of DNA damage, and repair associated proteins in DLB post-mortem brain tissue. Two major DNA damage repair pathways were highlighted via enrichment analysis, BER and DSB-repair via NHEJ with additional HR components also identified (Fig. 7 and Supplementary Table 6).

BER is activated as a consequence of alkylative, deaminative and oxidative base modification [3] and both abasic sites [27] and BER nucleases [87–90] have previously been reported as increased in synucleinopathy tissue. Here, upregulation of principle BER nucleases APE1 and FLAP endonuclease 1 (FEN1), is consistent with the overactivation of long patch BER, which can as a consequence of excessive nuclease activity, result in DSB formation rather than base repair [91–95]. Thus, the potential exists for BER overactivation in response to DLB pathology to contribute to the accumulation of DSBs. Proteins associated with NHEJ and HR repair pathways for DSB were also upregulated (Fig. 7b), including XRCC5 and XRCC6 also known as Ku70 and Ku80. Ku70 and Ku80 are the initial mediators of NHEJ [96] and are reported to be upregulated in a cell model of aSyn overexpression alongside the induction of DSBs [22].

Critically, the elevation of NHEJ and HR proteins does not appear to equate to an increased capacity for DSB repair as yH2AX remains increased in DLB cortical tissue. Indeed, prediction of any beneficial repair capacity enhancement following DDR protein upregulation is difficult, as isolated overexpression can be detrimental to cellular resilience against DNA damaging stressors [97]. Correspondingly, Ku80 and APE1 were recently reported as upregulated in a drosophila model of neurodegeneration, where their partial knockdown was protective [98]. Thus, such unregulated DDR activity may be a common degenerative pathway across the neurodegenerative spectrum. Nevertheless, in relation to DLB and other Lewy body diseases, functional studies are now required to establish the impact of this upregulation in the context of aSyn pathology.

## Conclusions

The present study highlights a prominent role for DNA damage in DLB pathology. We provide evidence for the seeding potential of nuclear extracts from DLB cases as well as the occurrence of DNA damage, specifically DSBs, within DLB cortical tissue. Such DSB accumulation is also present early in A30P mice and supports that the DSBs accumulation is as a consequence of the expression of dysfunctional mutant A30P aSyn. Moreover, we observed the cytoplasmic accumulation of genomic DNA damage markers within LBs. The data suggests a reciprocal relationship, whereby aSyn pathology induces DNA damage, which in turn generate ectopic cytoplasmic genomic material, capable of facilitating cytoplasmic aSyn aggregation. Cellular pathology occurred alongside marked upregulation of DDR, UPS and EIF3 complex proteins. Observed changes in nuclear proteome are supportive of disease-induced genomic instability. The overexpression and potentially overactivation of selective DDR components, may perturb repair, inducing additional damage. Despite the progress in delineating the occurrence of DNA damage and DDR alteration in DLB, it remains to be determined where within these pathways, nuclear aSyn may interact and the specific functional significance of the selective changes in DDR components. Nevertheless, the established early occurrence of DNA damage and the delineation of disease dependent DDR changes, highlights DNA damage and its repair as prominent targets for future therapeutic development.

## Supplementary Information

Supplementary Material 1. Supplementary table 1. Individual details for human tissue cohort. Each case used is listed with an arbitrary case number (Case no) and sex, age (years), post-mortem delay (PMD, hrs), Braak stage, Thal phase, Consortium to Establish a Registry for Alzheimer’s Disease (CERAD), the National Institute of Ageing – Alzheimer’s Association (NIA-AA) criteria, Lewy body (LB) Braak stage and McKeith criteria detailed. Additionally, tissue use for histology A (Hisa-XRCC1+γH2AX), histology B (Histb- γH2AX+pS129), Western blot (West), ELISA + seed amplification assay (SAA) and Mass spectrometry (MS) is also provided is also provided. NA = not available.

Supplementary Material 2. Supplementary table 2. Proteomic detection list and abundance analysis. Proteins identified as per the UniProt database and subsequently confirmed to be present with >2 unique peptides as well as being detected in >70% of samples are listed. For each protein, Entrez gene ID, gene name, individual Z-scores within batches (B1-3) for each sample are provided as well as pooled difference (DLB-Con), -Log(*p*-value) and significance (FDR<0.1). + = significant. Supplementary table 3. DAVID UniProt Keywords. Significantly enriched clusters from DAVID uniport keywords analysis. Enrichment database category, Keyword number, Keyword term, number of proteins present in upregulated list (count), % coverage of enrichment term, *p* value, associated genes and protein names are provided. Additionally detected term associated proteins within the background reference list (List Total), total proteins associated with the term (Pop Total), the fold enrichment, FDR adjustment and-Log10FDR values are provided. Supplementary table 4. DAVID GO Terms. Significantly enriched clusters from David GO terms analysis. Enrichment database category, Go number, term name, number of proteins present in upregulated list (count), % coverage of enrichment term, p value, associated genes and protein names are provided. Additionally detected term associated proteins within the background reference list (List Total), total proteins associated with the term (Pop Total), the fold enrichment, FDR adjustment and -Log10FDR values are provided. Supplementary table 5. STRING database GO term analysis of Cellular response to DNA damaging stimulus. All GO terms mapped to those proteins significantly upregulated which are associated with cellular response to DNA damaging stimulus. term ID, description, those upregulated proteins associated with the term (observed gene count), those present in the background list (Background gene count), strength of enrichment, FDR and matching protein ID and names are provided. Supplementary table 6. STRING database identified proteins and their associated DNA damage repair function. Identified proteins are listed with Gene symbol, Entrez gene ID, Gene names, an indication of their involvement in DNA damage repair pathways, a suggested DDR function and supportive citations. For DDR pathways, base-excision repair (BER), Double strand break repair (DSB), Non-homologous end joining (NHEJ) and homologous recombination (HR) as well as general role in repair is provided.

Supplementary Material 3. Supplementary Figure 1. All case traces of Thioflavin T fluorescence from seed amplification assay Mean fluorescence traces, established per control (Con) and dementia with Lewy body (DLB) cases, from triplicate runs. Note that all but one con case no appreciable increase in fluorescence over the 12 0hour time course is seen.

Supplementary Material 4. Supplementary figure 2. Neuropathological burden and correlation with yH2AX. a) Photomicrograph illustrating bespoke thresholds used to capture immunopositive signals from the lateral temporal cortex. Shown is i) Lewy pathology (Lewy bodies and Lewy neurites) stained with aSyn antibody KM51, ii) extracellular Aβ plaques stained with 4G8 and iii) phospho-tau stained with AT8 . Below transmission images, applied thresholds (red) are demonstrated. b) Quantification of area of images immunoreactive for i) Lewy pathology, ii) Aβ plaques and iii) phospho-tau, compared between control (Con) and DLB cases. c) Correlative (Spearman’s r) analysis tables of yH2AX values (as in main Fig 2) and quantified neuropathological burden and d) neuropathological assessment scales significant positive (red) and negative (blue) correlations highlighted. e) Plot of yH2AX and Lewy pathology coverage (KM51) for non-neuronal cells from DLB cases. *=*p*<0.05, ***=*p*<0.001, N.S = not significant, - = analysis not possible as all values 0. Scale bar in (a) represents 100mm and is valid for all images.

Supplementary Material 5. Supplementary Figure 3. All western blots for yH2AX. All blots used for western blot quantification of yH2AX. Nuclear extracts from control (Con) and dementia with Lewy body (DLB) cases were processed in 4 separate blots, normalised to histone H3 (H3) loading controls and normalised to mean control values within blots, prior to being pooled between blots. Note that blot 1 was developed in full with molecular weight marker to ensure specificity of signal. All other blots were cut prior to antibody staining.

Supplementary Material 6. Supplementary Figure 4. WT and A30P mice full data set including outlier. Identification of statistical outlier with A30P mice was established via Grubbs test as highlighted by red circled data point or arrow. Data is shown for a) Quantification of neuronal (NeuN +) and non-neuronal (NeuN -) mean γH2AX, b) XRCC1 and c) cell count of NeuN+ and NeuN - nuclei signal in WT (*n*=5) and A30P mice (*n*=5). Data are expressed in scatterplots with mean ± SEM (in b, c and f), $ =*p*<0.05 in Grubbs outlier test.

Supplementary Material 7. Supplementary Figure 5. High magnification images of cortical Lewy bodies enriched with yH2AX. Example images from two separate cases of Lewy bodies with colocalised yH2AX immunoreactivity. Shown are individual images from Z-stack LB capture shown is pS129-aSyn, yH2AX and a merged images in a single plane and a Z-project image as per maximal intensity with pS129-aSyn, yH2AX and DAPI co-stain. Orthogonal images shown for single plane merge images, further clarifying the co-localisation of yH2AX within LBs. Scale bar = 5µm.

Supplementary Material 8. Supplementary Figure 6. High magnification images of cortical Lewy bodies enriched with H3K27me3. Example images from two separate cases of aSyn aggregates with colocalised H3K27me3 immunoreactivity. Shown is individual images from z-stack, shown is pS129-aSyn, H3K27me3 and a merged images in a single plane and a Z-project image as per maximal intensity with pS129-aSyn, H3K27me3 and DAPI co-stain. Orthogonal images shown for single plane merge images, further clarifying the co-localisation of H3K27me3 within aSyn cytoplasmic aggregates. Scale bar = 5µm.

Supplementary Material 9. Supplementary Figure 7. Protein labelled heat map of altered nuclear proteins in DLB cases. Heat map of individual proteins abundance (as per within batch Z-scores). Each columns represents an individual case of either control (Con) or Dementia with Lewy bodies (DLB). Proteins are grouped as per those significantly upregulated and those significantly downregulated and arranged as per largest increase in abundance in DLB cases compared to controls.

Supplementary Material 10. Supplementary Figure 8. Gene Ontology analysis of upregulated nuclear proteins. Go term cluster enrichment of the 83 proteins established as elevated in nuclear proteome of DLB compared to controls. Fold enrichment and significance of individual terms are shown as per -log false discovery rate (FDR). Keywords are grouped into clusters as reported via the DAVID bioinformatic database. Dotted lines denote the boundaries of clusters, with cluster enrichment (C.Enrich) from background reference database shown within each cluster.

Supplementary Material 11. Supplementary Figure 9. Protein interaction map of upregulated nuclear proteins with extended cellular function clusters. STRING map of protein interactome of upregulated proteins with prominent functional category of “Cellular response to DNA damaging stimulus” (red), “Cellular macromolecule catabolic processes” (blue) and “Ribonucleoprotein complex” (green) highlighted. Individual proteins associated with each term denoted by colour. Physical and functional interactions are shown with the weighting of line indicating confidence in interaction.

Supplementary Material 12.

## Data Availability

All raw data is available upon request to corresponding authors. Additionally mass-spectrometry raw data is available at MassIVE, Centre for Computational Mass Spectrometry. Submission ID MSV000095358.

## Abbreviations

aSyn: Alpha-Synuclein
ATR: Ataxia telangiectasia and Rad3-related
ATM: Ataxia-telangiectasia mutated
AD: Alzheimer’s disease
ALS: Amyotrophic lateral Sclerosis
APEX1/APE: Apurinic/apyrimidinic endodeoxytibonucelease 1
BER: Base excision repair
BRCA1: Breast cancer gene 1
BSA: Bovine serum albumin
BA: Brodmann’s area
CDC5L: Cell division cycle 5 like
CERAD: Consortium to Establish a Registry for Alzheimer’s disease
Con: Control
CytoDNA: Cytoplasmic DNA
DDX1: DEAD-box helicase 1
DLB: Dementia with Lewy bodies
DDR: DNA damage repair
DNA-PK: DNA–protein kinase
DSB: Double strand break
EIF: Eukaryotic translation initiation factor
FEN1: Flap structure-specific endonuclease 1
GO: Gene Ontology
HDGFL2: Hepatoma-derived growth factor-like 2
HR: Homologous recombination
LB: Lewy Body
MRE11: Meiotic recombination 11
NIA-AA: National institute of ageing- Alzheimer’s association
NeuN: Neuronal nuclear protein
NHEJ: Non-Homologous end-joining
NGS: Normal goat serum
PFA: Paraformaldehyde
PBS: Phosphate buffered saline
PBST: Phosphate buffered saline with Tween 20
RAD51: Radiation sensitive protein 51
RPA1: Replication protein A1
RBBP8/CtIP: Retinoblastoma binding protein 8
RNF: Ring finger
53BP1: P53 binding protein 1
PD: Parkinson’s disease
PCNA: Proliferating nuclear protein A
PSMD14: Proteasome 26S subunit, non-ATPase 14
PPI: Protein–protein interaction
PARP1: Poly (ADP) Ribose polymerase 1
ps129: Phosphorylated serine 129
SAA: Seed amplification assay
SSB: Single strand break
ThT: Thioflavin T
TBS: Tris-buffered saline
TBST: Tris-buffered saline with Tween-20
UPS: Ubiquitin proteasome system
WT: Wild type
XRCC: X-ray repair cross complementing
