# Supplementary material for: A reciprocal relationship between markers of genomic DNA damage and alpha-synuclein pathology in dementia with Lewy bodies
Source: Mol Neurodegener. 2025 Mar 20;20:34. doi: 10.1186/s13024-025-00813-4 (PMC11927131; doi:10.1186/s13024-025-00813-4)
Supplement: Supplementary file 1 — Supplementary Material 1. Supplementary table 1. Individual details for human tissue cohort. Each case used is listed with an arbitrary case number (Case no) and sex, age (years), post-mortem delay (PMD, hrs), Braak stage, Thal phase, Consortium to Establish a Registry for Alzheimer’s Disease (CERAD), the National Institute of Ageing – Alzheimer’s Association (NIA-AA) criteria, Lewy body (LB) Braak stage and McKeith criteria detailed. Additionally, tissue use for histology A (Hisa-XRCC1+γH2AX), histology B (Histb- γH2AX+pS129), Western blot (West), ELISA + seed amplification assay (SAA) and Mass spectrometry (MS) is also provided is also provided. NA = not available. [file 13024_2025_813_MOESM1_ESM.docx]

| Case nr | Diagnosis | age | sex | PMI | NFT Braak | Thal Phase | CERAD | NIA-AA | LB Braak | McKeith | Usage |
| --- | --- | --- | --- | --- | --- | --- | --- | --- | --- | --- | --- |
| 1 | Con | 88 | F | 22 | III | 0 | 0 | No | 0 | No | Hisa+b+West |
| 2 | Con | 60 | M | 60 | 0 | 1 | 0 | Low | 0 | No | Hisa+b+MS+West |
| 3 | Con | 88 | M | 24 | II | 3 | 0 | Low | 0 | No | Hisa+b+MS+West |
| 4 | Con | 85 | M | 45 | III | 4 | 1 | Inter | 0 | No | Hisa+b+MS+West |
| 5 | Con | 90 | F | 63 | II | 0 | 0 | No | 0 | No | Hisa+b+West |
| 6 | Con | 73 | M | 25 | 0 | 0 | 0 | No | 0 | No | Hisa+b+MS+West + ELSIA + SAA |
| 7 | Con | 72 | M | 60 | II | 5 | 0 | Low | 0 | No | Hisa+b+MS+West |
| 8 | Con | 95 | F | 66 | III | 0 | 0 | No | 0 | No | Hisa+b+West + ELISA + SAA |
| 9 | Con | 80 | F | 25 | II | 1 | 0 | Low | 0 | No | Hisa+b +MS+ West + ELSIA + SAA |
| 10 | Con | 94 | F | 15 | II | 1 | 0 | Low | 0 | No | Hisa+b + West |
| 11 | Con | 81 | M | 43 | II | 0 | 0 | No | 0 | No | Hisa+b+West + ELSIA + SAA |
| 12 | Con | 66 | M | 56 | 0 | 2 | 0 | Low | 0 | No | Hisa+b+MS+West |
| 13 | Con | 99 | F | 5 | II | 0 | 0 | No | 0 | No | Hisa+MS+West |
| 14 | Con | 74 | F | 74 | III | 0 | 0 | No | 0 | No | MS+West |
|  |  |  |  |  |  |  |  |  |  |  |  |
| 15 | DLB | 86 | M | 96 | III | 5 | 2 | Inter | 4 | Limbic | Hisa+b |
| 16 | DLB | 91 | F | 10 | III | 5 | 2 | Inter | 6 | Neo | Hisa+b + ELISA + SAA + West |
| 17 | DLB | 77 | M | 24 | III | 2 | 0 | Inter | 6 | Neo | Hisa+b+MS+West |
| 18 | DLB | 76 | M | 13 | II | 0 | 0 | Low | NA | Neo | Hisa+b+MS+West |
| 19 | DLB | 72 | M | 89 | III | 0 | 0 | No | 6 | Neo | Hisa+b+West + ELISA + SAA |
| 20 | DLB | 77 | M | 8 | II | 0 | 0 | No | NA | Neo | Hisa+b+MS+West |
| 21 | DLB | 81 | M | 21 | III | 4 | 1 | Inter | 6 | Neo | Hisa+b+West |
| 22 | DLB | 75 | M | 18 | II | NA | NA | NA | NA | Limbic | Hisa+b+MS+West |
| 23 | DLB | 81 | M | 26 | III | 3 | 2 | Inter | 6 | Neo | Hisa+b +MS+West + ELISA + SAA |
| 24 | DLB | 71 | M | 68 | II | NA | NA | NA | NA | Neo | Hisa+b+MS+West |
| 25 | DLB | 73 | M | 47 | III | 1 | 0 | Low | 6 | Neo | Hisa+b+West + ELISA + SAA |
| 26 | DLB | 82 | M | 46 | II | 3 | 0 | Low | 6 | Neo | Hisa+b+MS+West |
| 27 | DLB | 78 | M | 8 | III | 4 | 2 | Inter | 6 | Neo | MS+West |
| 28 | DLB | 80 | M | 34 | III | 4 | 1 | Low | 6 | Neo | MS+West |

Supplementary table 1. List of cases within research cohort.
