# Supplementary figures and images for: A reciprocal relationship between markers of genomic DNA damage and alpha-synuclein pathology in dementia with Lewy bodies
Source: Mol Neurodegener. 2025 Mar 20;20:34. doi: 10.1186/s13024-025-00813-4 (PMC11927131; doi:10.1186/s13024-025-00813-4)

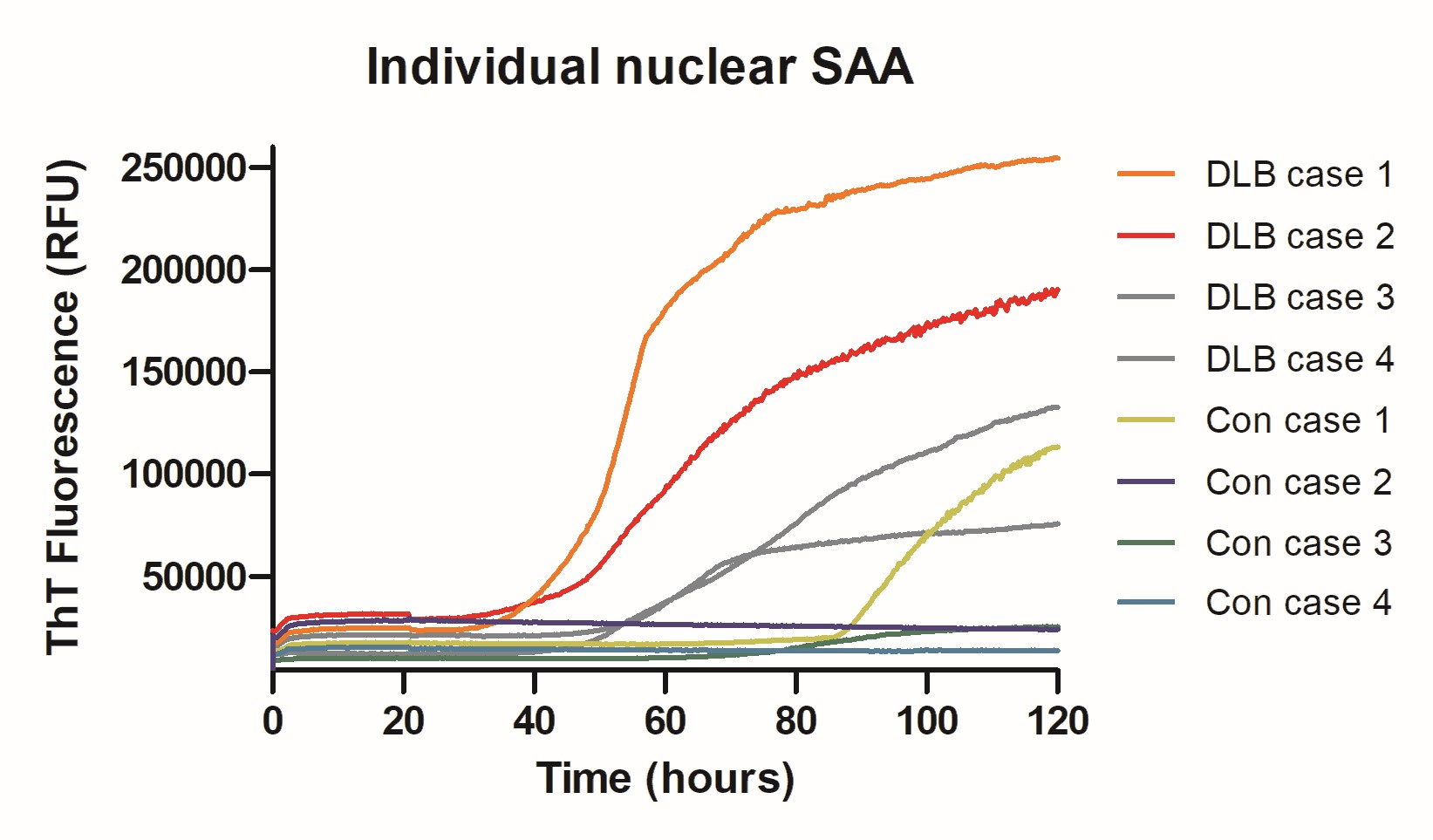

Supplement: Supplementary file 3 — Supplementary Material 3. Supplementary Figure 1. All case traces of Thioflavin T fluorescence from seed amplification assay Mean fluorescence traces, established per control (Con) and dementia with Lewy body (DLB) cases, from triplicate runs. Note that all but one con case no appreciable increase in fluorescence over the 12 0hour time course is seen. [file 13024_2025_813_MOESM3_ESM.tif]

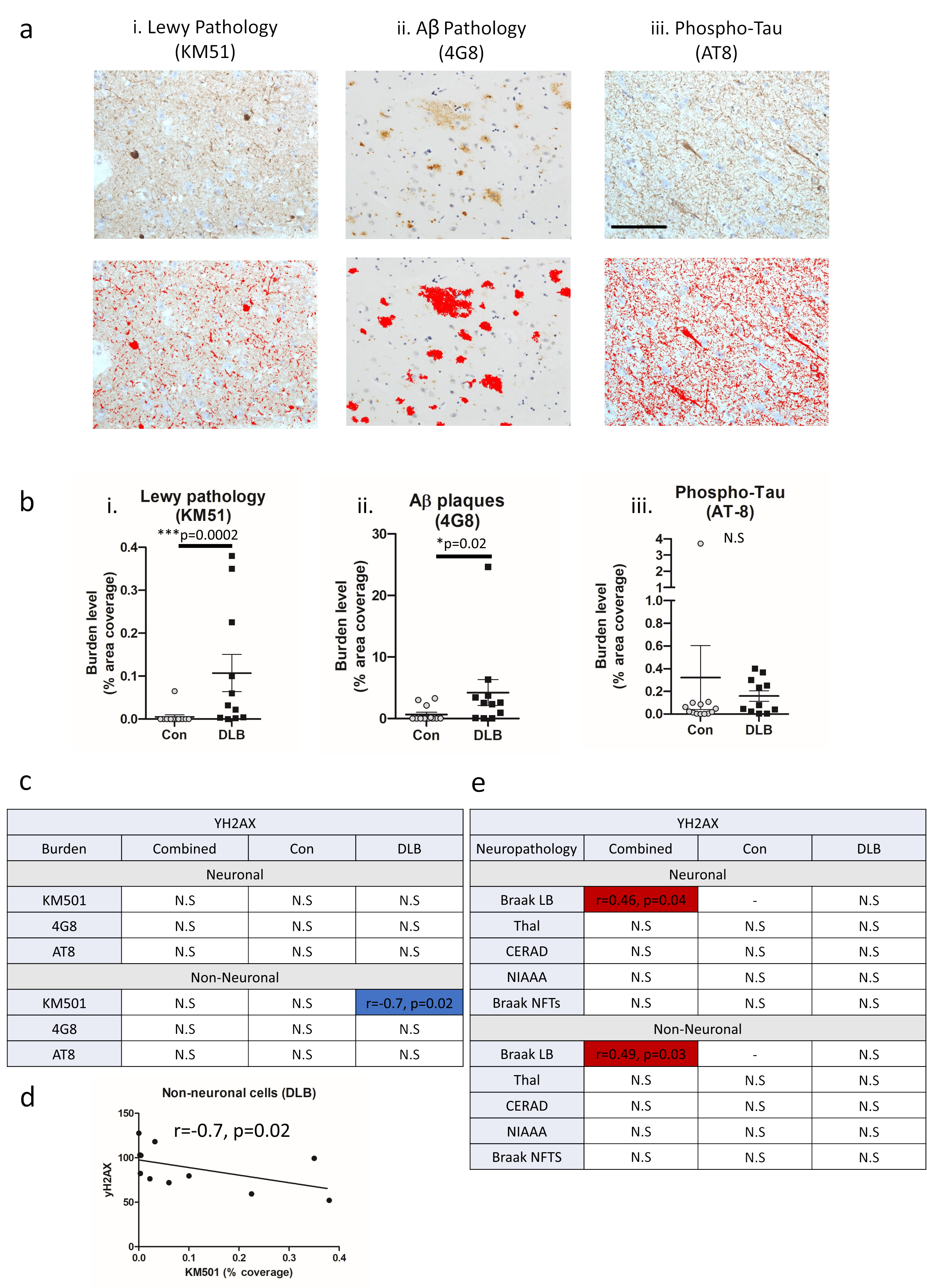

Supplement: Supplementary file 4 — Supplementary Material 4. Supplementary figure 2. Neuropathological burden and correlation with yH2AX. a) Photomicrograph illustrating bespoke thresholds used to capture immunopositive signals from the lateral temporal cortex. Shown is i) Lewy pathology (Lewy bodies and Lewy neurites) stained with aSyn antibody KM51, ii) extracellular Aβ plaques stained with 4G8 and iii) phospho-tau stained with AT8 . Below transmission images, applied thresholds (red) are demonstrated. b) Quantification of area of images immunoreactive for i) Lewy pathology, ii) Aβ plaques and iii) phospho-tau, compared between control (Con) and DLB cases. c) Correlative (Spearman’s r) analysis tables of yH2AX values (as in main Fig 2) and quantified neuropathological burden and d) neuropathological assessment scales significant positive (red) and negative (blue) correlations highlighted. e) Plot of yH2AX and Lewy pathology coverage (KM51) for non-neuronal cells from DLB cases. *=p<0.05, ***=p<0.001, N.S = not significant, - = analysis not possible as all values 0. Scale bar in (a) represents 100mm and is valid for all images. [file 13024_2025_813_MOESM4_ESM.tif]

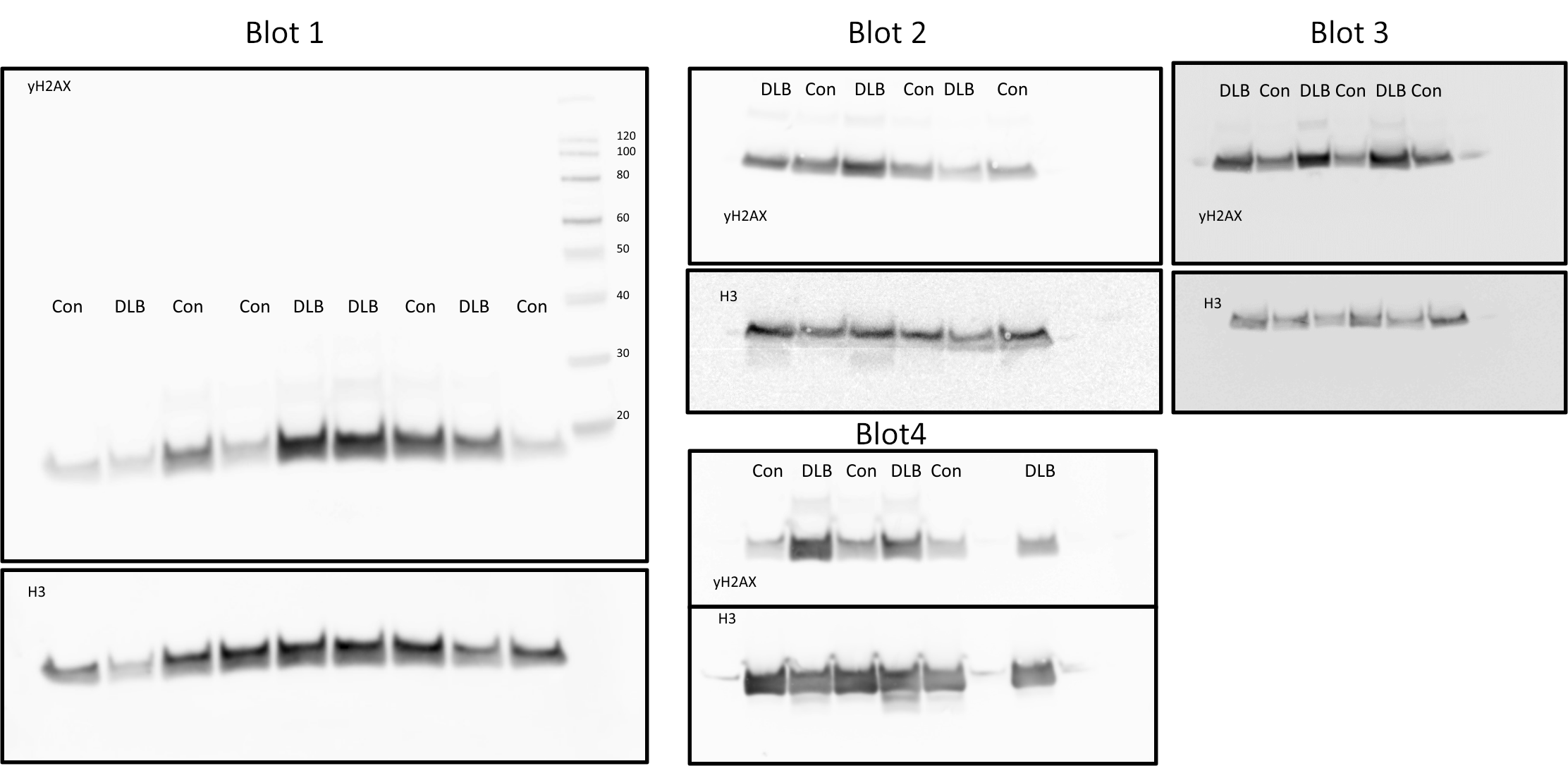

Supplement: Supplementary file 5 — Supplementary Material 5. Supplementary Figure 3. All western blots for yH2AX. All blots used for western blot quantification of yH2AX. Nuclear extracts from control (Con) and dementia with Lewy body (DLB) cases were processed in 4 separate blots, normalised to histone H3 (H3) loading controls and normalised to mean control values within blots, prior to being pooled between blots. Note that blot 1 was developed in full with molecular weight marker to ensure specificity of signal. All other blots were cut prior to antibody staining. [file 13024_2025_813_MOESM5_ESM.tif]

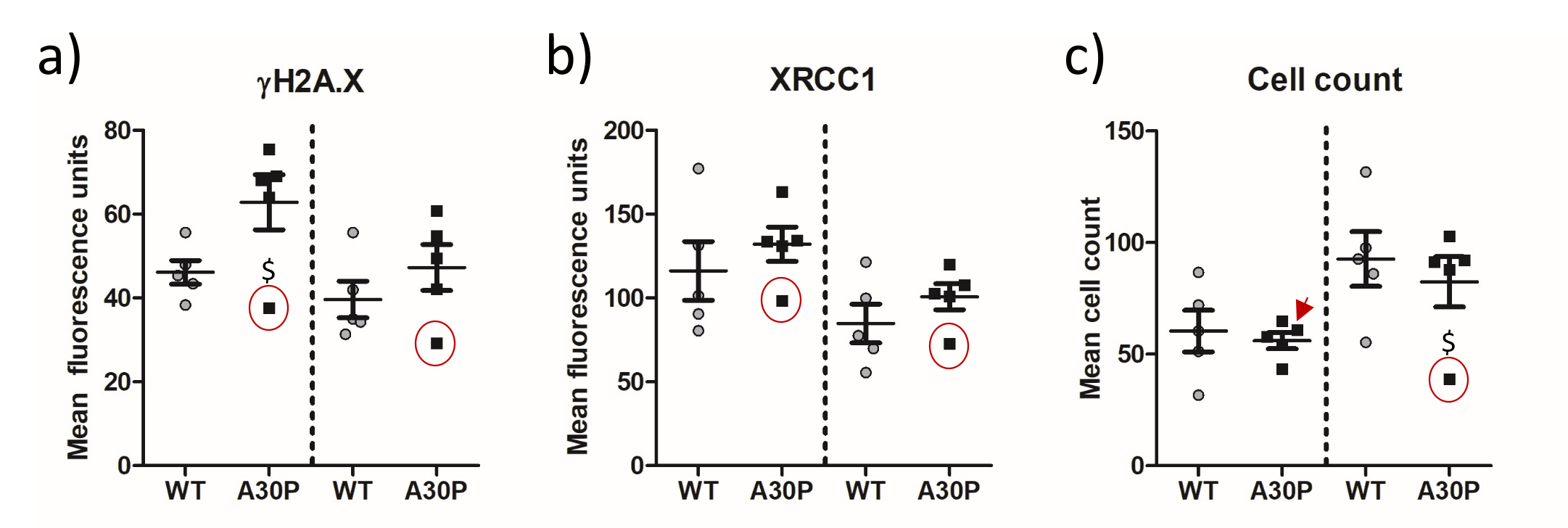

Supplement: Supplementary file 6 — Supplementary Material 6. Supplementary Figure 4. WT and A30P mice full data set including outlier. Identification of statistical outlier with A30P mice was established via Grubbs test as highlighted by red circled data point or arrow. Data is shown for a) Quantification of neuronal (NeuN +) and non-neuronal (NeuN -) mean γH2AX, b) XRCC1 and c) cell count of NeuN+ and NeuN - nuclei signal in WT (n=5) and A30P mice (n=5). Data are expressed in scatterplots with mean ± SEM (in b, c and f), $ =p<0.05 in Grubbs outlier test. [file 13024_2025_813_MOESM6_ESM.tif]

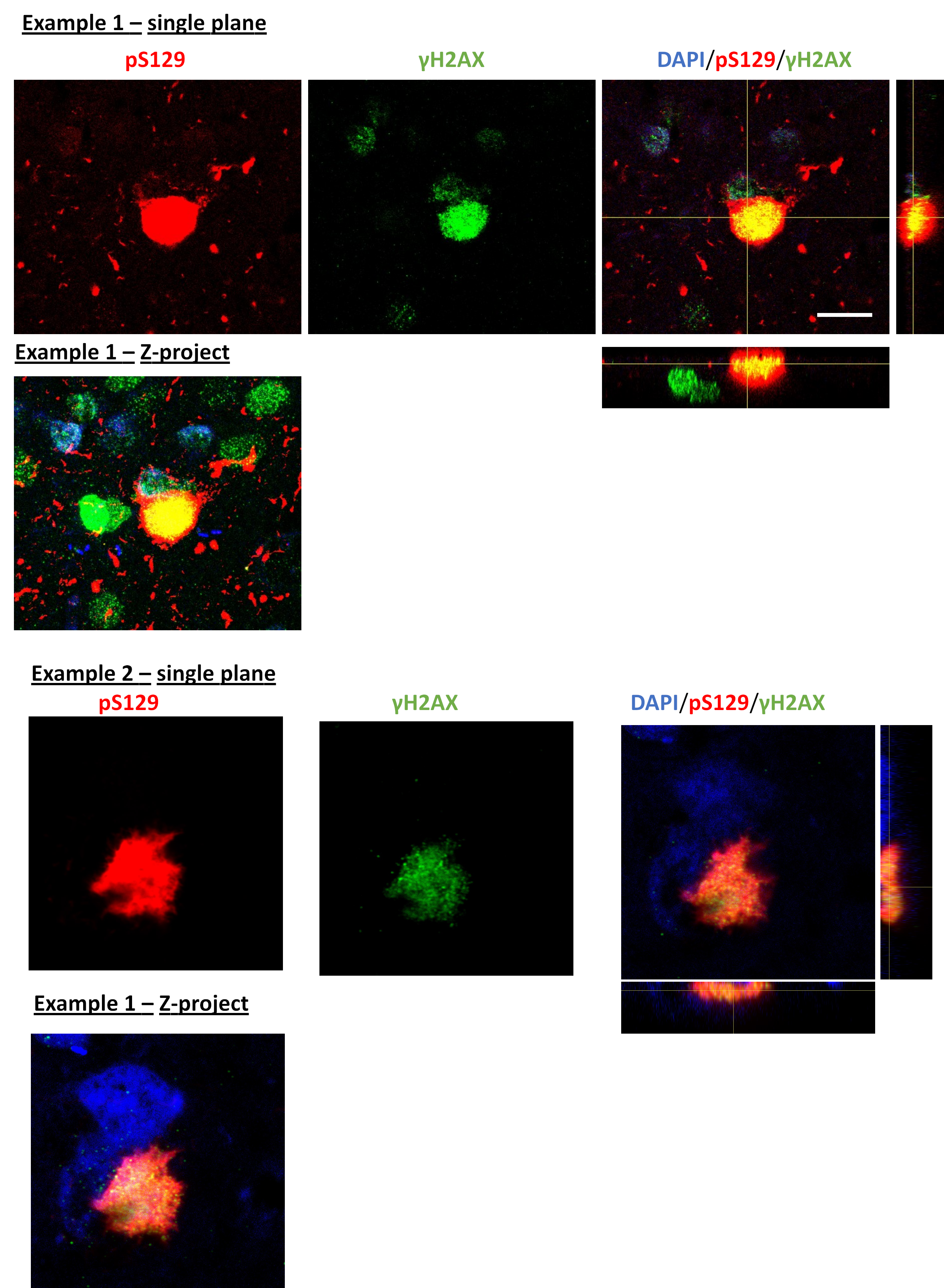

Supplement: Supplementary file 7 — Supplementary Material 7. Supplementary Figure 5. High magnification images of cortical Lewy bodies enriched with yH2AX. Example images from two separate cases of Lewy bodies with colocalised yH2AX immunoreactivity. Shown are individual images from Z-stack LB capture shown is pS129-aSyn, yH2AX and a merged images in a single plane and a Z-project image as per maximal intensity with pS129-aSyn, yH2AX and DAPI co-stain. Orthogonal images shown for single plane merge images, further clarifying the co-localisation of yH2AX within LBs. Scale bar = 5µm. [file 13024_2025_813_MOESM7_ESM.tif]

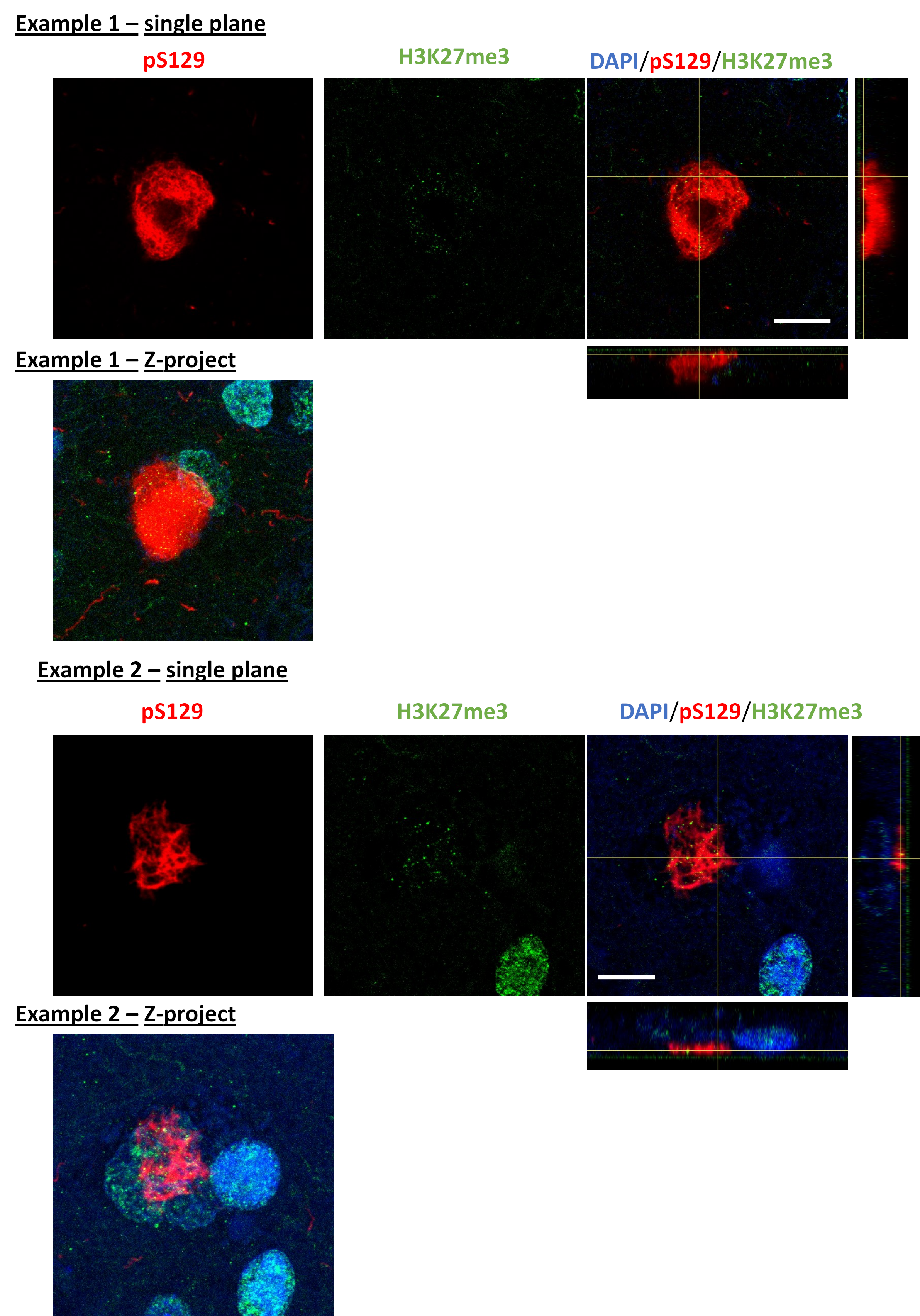

Supplement: Supplementary file 8 — Supplementary Material 8. Supplementary Figure 6. High magnification images of cortical Lewy bodies enriched with H3K27me3. Example images from two separate cases of aSyn aggregates with colocalised H3K27me3 immunoreactivity. Shown is individual images from z-stack, shown is pS129-aSyn, H3K27me3 and a merged images in a single plane and a Z-project image as per maximal intensity with pS129-aSyn, H3K27me3 and DAPI co-stain. Orthogonal images shown for single plane merge images, further clarifying the co-localisation of H3K27me3 within aSyn cytoplasmic aggregates. Scale bar = 5µm. [file 13024_2025_813_MOESM8_ESM.tif]

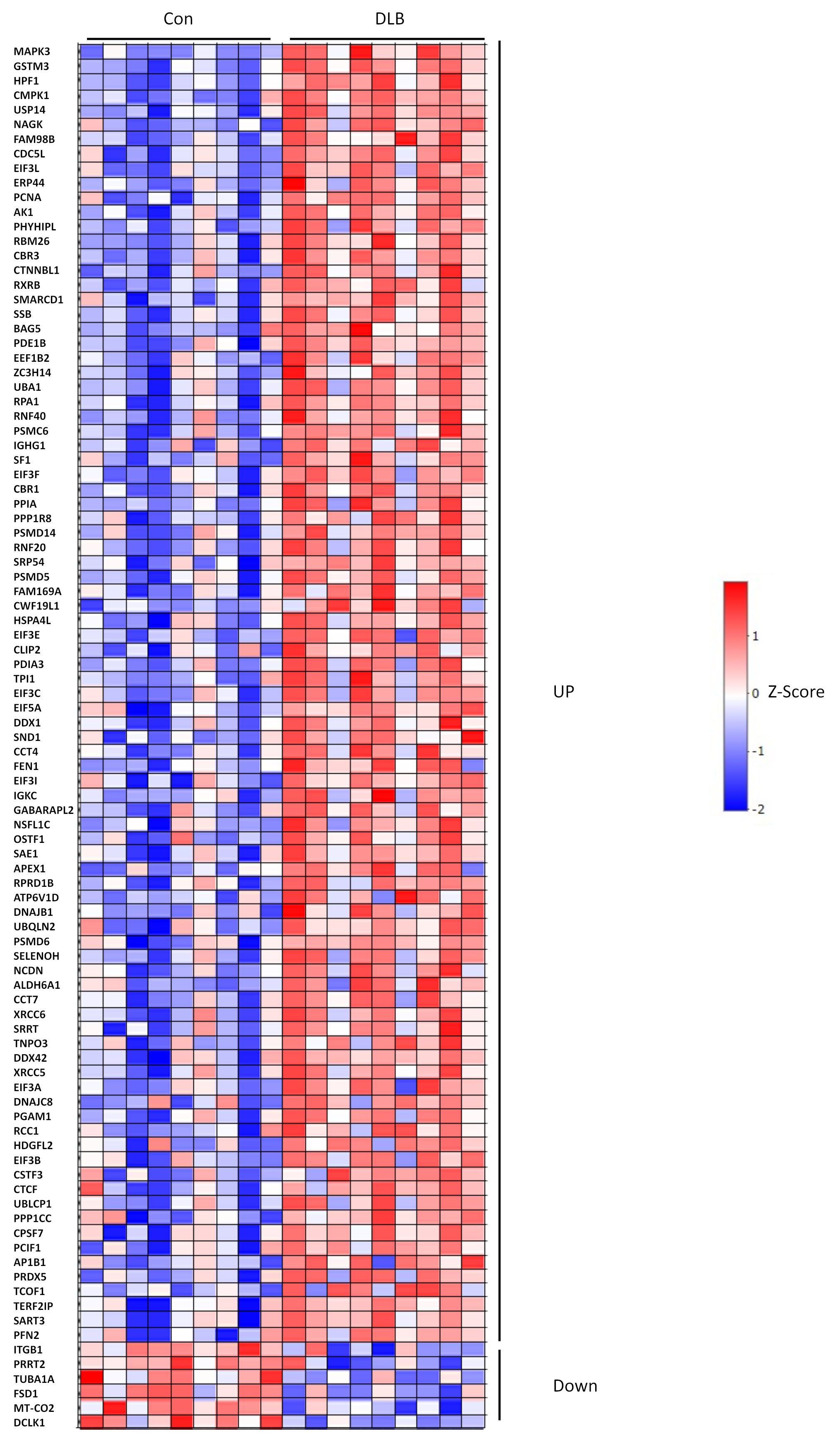

Supplement: Supplementary file 9 — Supplementary Material 9. Supplementary Figure 7. Protein labelled heat map of altered nuclear proteins in DLB cases. Heat map of individual proteins abundance (as per within batch Z-scores). Each columns represents an individual case of either control (Con) or Dementia with Lewy bodies (DLB). Proteins are grouped as per those significantly upregulated and those significantly downregulated and arranged as per largest increase in abundance in DLB cases compared to controls. [file 13024_2025_813_MOESM9_ESM.tif]

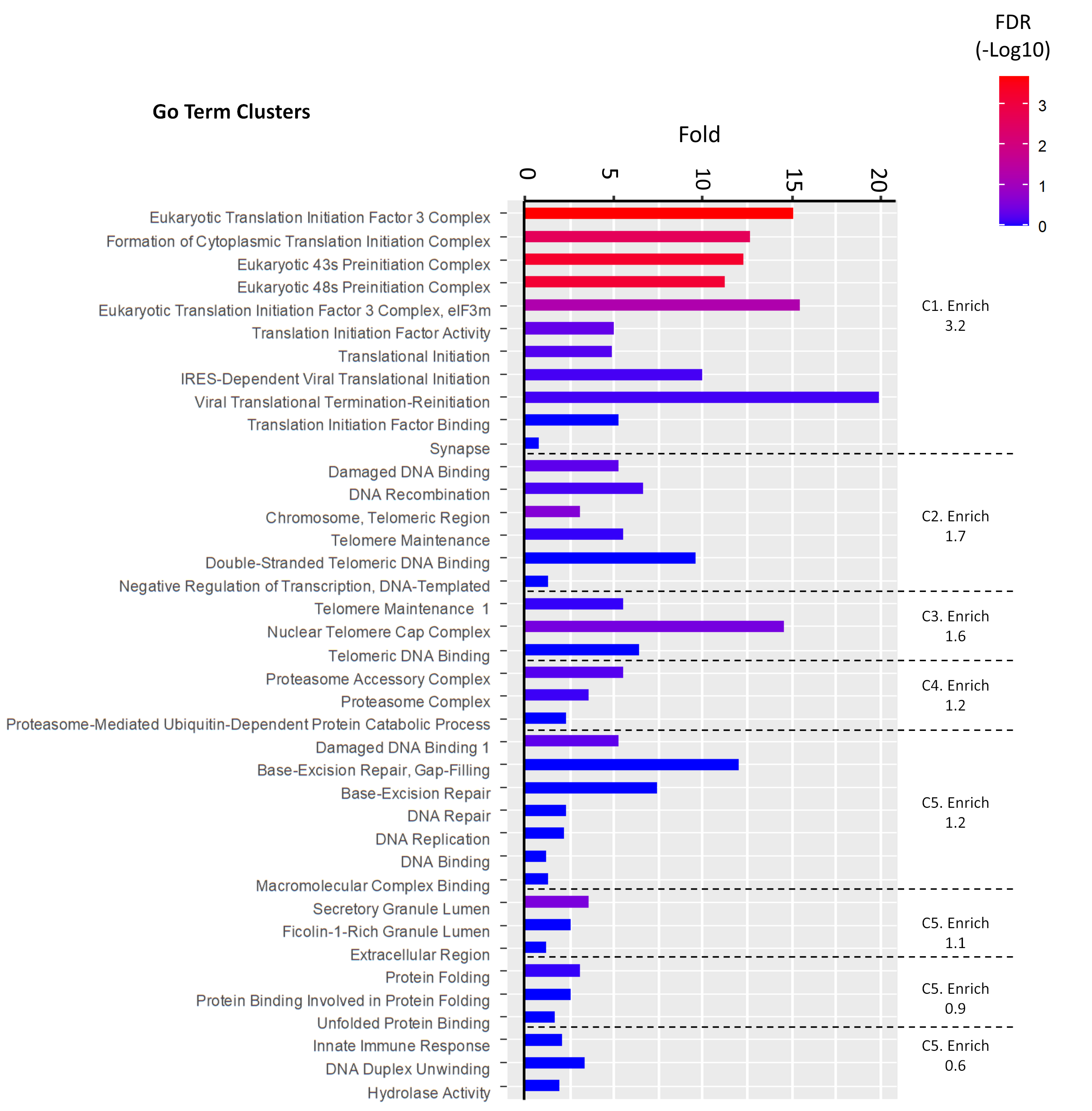

Supplement: Supplementary file 10 — Supplementary Material 10. Supplementary Figure 8. Gene Ontology analysis of upregulated nuclear proteins. Go term cluster enrichment of the 83 proteins established as elevated in nuclear proteome of DLB compared to controls. Fold enrichment and significance of individual terms are shown as per -log false discovery rate (FDR). Keywords are grouped into clusters as reported via the DAVID bioinformatic database. Dotted lines denote the boundaries of clusters, with cluster enrichment (C.Enrich) from background reference database shown within each cluster. [file 13024_2025_813_MOESM10_ESM.tif]

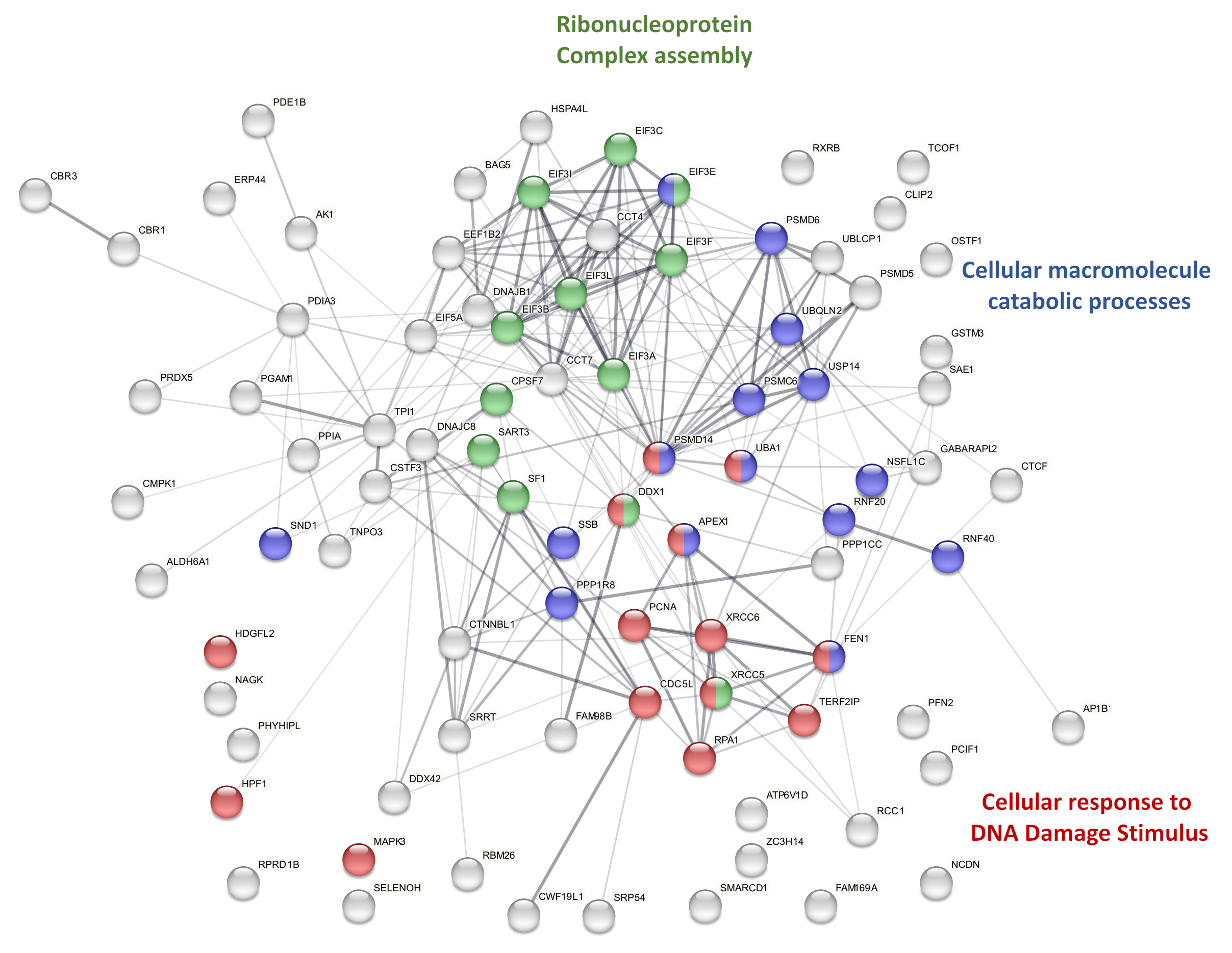

Supplement: Supplementary file 11 — Supplementary Material 11. Supplementary Figure 9. Protein interaction map of upregulated nuclear proteins with extended cellular function clusters. STRING map of protein interactome of upregulated proteins with prominent functional category of “Cellular response to DNA damaging stimulus” (red), “Cellular macromolecule catabolic processes” (blue) and “Ribonucleoprotein complex” (green) highlighted. Individual proteins associated with each term denoted by colour. Physical and functional interactions are shown with the weighting of line indicating confidence in interaction. [file 13024_2025_813_MOESM11_ESM.tif]
