# Supplementary material for: A reciprocal relationship between markers of genomic DNA damage and alpha-synuclein pathology in dementia with Lewy bodies
Source: Mol Neurodegener. 2025 Mar 20;20:34. doi: 10.1186/s13024-025-00813-4 (PMC11927131; doi:10.1186/s13024-025-00813-4)
Supplement: Supplementary file 12 — Supplementary Material 12. [file 13024_2025_813_MOESM12_ESM.docx]

Supplemental methods

Batch processing of temporal cortex nuclear extracts. Batches were conducted with 3 control and 3 DLB cases in each, with slight variation in sample preparation. Frozen samples (250mg) were fractioned as described in main text and nuclear pellets collected.

**Batch 1**

Samples were mixed in 1:1 ratio with 10% SDS in 100mM Triethylammonium bicarbonate (TEAB) pH 8.5, heated to 95oC and sonicated to clear of DNA/ RNA.. Protein concentration was measured using Pierce™ BCA Protein Assay Kit (Thermo Scientific™). Proteins were reduced TCEP (final concentration of 5mM, 55 °C for 15min), cysteines alkylated by incubation with MMTS (20mM final concentration, at RT, 10 min) and with 12% Phosphoric acid to a final concentration of 2.5% (v/v).

Protein digestion was carried out with S-Trap micro spin columns (Protifi). The samples were loaded onto spin columns in 6 volumes of binding buffer (90% methanol 100mM TEAB pH 7.55) and centrifuged at 4000xg for 30s. The columns were then washed with binding buffer (three times) and the flow through discarded. Proteins were digested with trypsin (Worthington) in 50mM TEAB pH 8.5, at a ratio of 10:1 protein to trypsin, for 3h at 47oC.

Peptides were eluted with three washes of the trap; first 50 ul 50mM TEAB, second 50ul 0.2% formic acid and third 50 ul 50% acetonitrile with 0.2% formic acid. The solution was frozen then dried in a centrifugal concentrator and reconstituted in 0.2% formic acid to a concentration of 500ng/ul.

Equivalents of 0.5ug of each peptide sample were loaded per LCMS run (using an UltiMate 3000 RSLCnano HPLC), first onto a 300μm x 5mm C18 PepMap C18 trap cartridge (Thermo Fisher Scientific) in 0.1% formic acid at 10 µl/min for 5min and then separated on separated on a 75μmx50cm C18 column (Thermo EasySpray -C18 2 µm) with integrated emitter, using a 110min gradient from 97 % A (0.1% FA in 3% DMSO) and 3% B (0.1% FA in 80% ACN 3% DMSO), to 35 % B, at a flow rate of 250nl/min. The separated peptides were then injected into Exploris 480 Quadrupole-Orbitrap Mass Spectrometer (Thermo Fisher Scientific, Waltham, MA, U.S.A.) via EasySpray source at the Ion Transfer Tube temperature of 280oC, spray voltage 1500 V and analysed using data dependent (DDA) acquisition. The total LCMS run time was 150min. Orbitrap full scan resolution was 120,000, RF lens 50%, ACG Target set to “Standard”, Scan Range 400-1600m/z. Top 20 method was implemented to select precursors for MSMS. MIPS set to peptide, Intensity threshold 5.0 e3, charge state 2-7 and dynamic exclusion after 1 times for 35 s 10ppm mass tolerance. ddMS2 scans were performed at 15000 resolution, HCD collision energy 30%, first mass 110 m/z, ACG Target set to 100%.

**Batch 2**

Frozen samples (250 mg) were fractionated as above, nuclear pellets collected, resuspended with 5%SDS in PBS, then heated to 95oC and sonicated to clear of DNA/ RNA. Protein concentration was measured using Pierce™ BCA Protein Assay Kit (Thermo Scientific™).

Proteins were reduced with dithiothreitol (DTT) at a final concentration of 40 mM (65oC, 30min), cysteines alkylated by incubation with iodoacetamide (80 mM final concentration, 30min, room temp. in dark) and then acidified by adding 27.5% Phosphoric acid to a final concentration of 2.5% (v/v). Protein digestion was carried out with S-Trap micro spin columns (Protifi). The samples were loaded onto spin columns in 6 volumes of binding buffer (90% methanol 100mM TEAB pH 7.55) and centrifuged at 4000xg for 30s. The columns were then washed with binding buffer (three times) and the flow through discarded. Proteins were digested with trypsin (Worthington) in 50mM TEAB pH 8.5, at a ratio of 20:1 protein to trypsin, overnight at 37oC.

Peptides were eluted with three washes of the trap; first 50 ul 50mM TEAB, second 50ul 0.2% formic acid and third 50 ul 50% acetonitrile with 0.2% formic acid. The solution was frozen then dried in a centrifugal concentrator and reconstituted in 0.2% formic acid to a concentration of 1ug/ul.

Equivalents of 1ug of each peptide sample were loaded per LCMS run (using an UltiMate 3000 RSLCnano HPLC), first onto a 75μm x 20mm C18 Acclaim™ PepMap™ 100 C18 HPLC Trap Column (Thermo Fisher Scientific) in 0.1% formic acid at 10 µl/min for 5min and then separated on separated on a 75μmx25cm C18 column (Thermo EasySpray -C18 2 µm) with integrated emitter, using a 60min gradient from 97 % A (0.1% FA in 3% DMSO) and 3% B (0.1% FA in 80% ACN 3% DMSO), to 35 % B, at a flow rate of 400nl/min. The separated peptides were then injected into Exploris 480 Quadrupole-Orbitrap Mass Spectrometer (Thermo Fisher Scientific, Waltham, MA, U.S.A.) via EasySpray source at the Ion Transfer Tube temperature of 280oC, spray voltage 1900 V and analysed using data dependent (DDA) acquisition. The total LCMS run time was 90min. Orbitrap full scan resolution was 60,000, RF lens 50%, ACG Target set to 300%, Scan Range 400-1600m/z. Top 20 method was implemented to select precursors for MSMS. MIPS set to peptide, Intensity threshold 5.0 e3, charge state 2-7 and dynamic exclusion after 2 times for 35s, 10ppm mass tolerance. ddMS2 scans were performed at 15000 resolution, HCD collision energy 30%, first mass 110 m/z, ACG Target set to 100%.

**Batch 3**

Frozen samples (250 mg) were fractionated as above, nuclear pellets collected, resuspended with 5%SDS in PBS, then heated to 95oC and sonicated to clear of DNA/ RNA. Protein concentration was measured using Pierce™ BCA Protein Assay Kit (Thermo Scientific™). Proteins were reduced with dithiothreitol (DTT) at a final concentration of 40 mM (65oC, 30min), cysteines alkylated by incubation with iodoacetamide (80 mM final concentration, 30min, room temp. in dark) and then acidified by adding 27.5% Phosphoric acid to a final concentration of 2.5% (v/v).

Protein digestion was carried out with S-Trap micro spin columns (Protifi). The samples were loaded onto spin columns in 6 volumes of binding buffer (90% methanol 100mM TEAB pH 7.55) and centrifuged at 4000xg for 30s. The columns were then washed with binding buffer (three times) and the flow through discarded. Proteins were digested with trypsin (Worthington) in 50mM TEAB pH 8.5, at a ratio of 20:1 protein to trypsin, 90min. at 47oC.

Peptides were eluted with three washes of the trap; first 50ul 50mM TEAB, second 50ul 0.2% formic acid and third 50ul 50% acetonitrile with 0.2% formic acid. The solution was frozen then dried in a centrifugal concentrator and reconstituted in 0.2% formic acid to a concentration of 1ug/ul.

Equivalents of 1ug of each peptide sample were loaded per LCMS run (using an UltiMate 3000 RSLCnano HPLC), first onto a 75μm x 20mm C18 Acclaim™ PepMap™ 100 C18 HPLC Trap Column (Thermo Fisher Scientific) in 0.1% formic acid at 10 µl/min for 5min and then separated on separated on a 50 cm RP-C18 µPAC™ column (PharmaFluidics), using a 60min gradient from 97 % A (0.1% FA in 3% DMSO) and 3% B (0.1% FA in 80% ACN 3% DMSO), to 35 % B, at a flow rate of 400nl/min. The separated peptides were then injected into Exploris 480 Quadrupole-Orbitrap Mass Spectrometer (Thermo Fisher Scientific, Waltham, MA, U.S.A.) via Thermo Scientific μPAC compatible EasySpray emitter at the Ion Transfer Tube temperature of 280oC, spray voltage 1600 V and analysed using data dependent (DDA) acquisition. The total LCMS run time was 90min. Orbitrap full scan resolution was 120,000, RF lens 40%, ACG Target set to 300%, Scan Range 400-1600m/z. Top 20 method was implemented to select precursors for MSMS. MIPS set to peptide, Intensity threshold 5.0 e3, charge state 2-5 and dynamic exclusion after 1 times for 30s, 10ppm mass tolerance. ddMS2 scans were performed at 30000 resolution, HCD collision energy 30%, first mass 110 m/z, ACG Target set to 100%.
